# Estimating the annual dengue force of infection from the age of reporting primary infections across urban centres in endemic countries

**DOI:** 10.1186/s12916-021-02101-6

**Published:** 2021-09-30

**Authors:** Joseph R. Biggs, Ava Kristy Sy, Katharine Sherratt, Oliver J. Brady, Adam J. Kucharski, Sebastian Funk, Mary Anne Joy Reyes, Mary Ann Quinones, William Jones-Warner, Ferchito L. Avelino, Nemia L. Sucaldito, Amado O. Tandoc, Eva Cutiongco-de la Paz, Maria Rosario Z. Capeding, Carmencita D. Padilla, Julius Clemence R. Hafalla, Martin L. Hibberd

**Affiliations:** 1 Department of Infection Biology, Faculty of Infectious and Tropical Diseases, London School of Hygiene and Tropical Medicine, London, UK; 2 Department of Virology, Research Institute for Tropical Medicine, Manila, Philippines; 3 Dengue Study Group, Research Institute for Tropical Medicine, Manila, Philippines; 4 Department of Infectious Disease Epidemiology, Faculty of Epidemiology and Population Health, London School of Hygiene and Tropical Medicine, London, UK; 5 Centre for the Mathematical Modelling of Infectious Diseases, London School of Hygiene and Tropical Medicine, London, UK; 6 Department of Health, Philippine Epidemiology Bureau, Manila, Philippines; 7 Institute of Human Genetics, National Institute of Health, University of the Philippines, Manila, Philippines; 8 Philippine Genome Centre, University of the Philippines, Manila, Philippines

**Keywords:** Dengue, Surveillance, Serology, Primary, Flavivirus, Philippines

## Abstract

**Background:**

Stratifying dengue risk within endemic countries is crucial for allocating limited control interventions. Current methods of monitoring dengue transmission intensity rely on potentially inaccurate incidence estimates. We investigated whether incidence or alternate metrics obtained from standard, or laboratory, surveillance operations represent accurate surrogate indicators of the burden of dengue and can be used to monitor the force of infection (FOI) across urban centres.

**Methods:**

Among those who reported and resided in 13 cities across the Philippines, we collected epidemiological data from all dengue case reports between 2014 and 2017 (*N* 80,043) and additional laboratory data from a cross-section of sampled case reports (*N* 11,906) between 2014 and 2018. At the city level, we estimated the aggregated annual FOI from age-accumulated IgG among the non-dengue reporting population using catalytic modelling. We compared city-aggregated FOI estimates to aggregated incidence and the mean age of clinically and laboratory diagnosed dengue cases using Pearson’s Correlation coefficient and generated predicted FOI estimates using regression modelling.

**Results:**

We observed spatial heterogeneity in the dengue average annual FOI across sampled cities, ranging from 0.054 [0.036–0.081] to 0.249 [0.223–0.279]. Compared to FOI estimates, the mean age of primary dengue infections had the strongest association (*ρ* −0.848, *p* value<0.001) followed by the mean age of those reporting with warning signs (*ρ* −0.642, *p* value 0.018). Using regression modelling, we estimated the predicted annual dengue FOI across urban centres from the age of those reporting with primary infections and revealed prominent spatio-temporal heterogeneity in transmission intensity.

**Conclusions:**

We show the mean age of those reporting with their first dengue infection or those reporting with warning signs of dengue represent superior indicators of the dengue FOI compared to crude incidence across urban centres. Our work provides a framework for national dengue surveillance to routinely monitor transmission and target control interventions to populations most in need.

**Supplementary Information:**

The online version contains supplementary material available at 10.1186/s12916-021-02101-6.

## Background

Dengue is a mosquito-borne viral disease in which individuals can suffer up to four times during their lifetime due to the existence of four distinct serotypes (DENV1-4). A primary (first) dengue infection with any serotype induces IgG antibodies that offer protection against homologous serotype infections yet allow subsequent post-primary (secondary, tertiary or quaternary) infections with heterologous serotypes [1, 2]. Severe dengue disease is associated with, though not limited to, secondary infections due to pre-existing, cross-reactive, IgG antibodies that do not protect against infection, but rather facilitate antibody-dependent enhancement of viral replication [3]. Without specific therapeutics or a widely available vaccine, costly vector control interventions remain the predominant method of dengue control [4]. To appropriately deploy these limited interventions, dengue national surveillance operations in endemic countries must accurately monitor force of infection (FOI) over space and time.

In countries where dengue is a notifiable disease, national surveillance efforts typically collate dengue case reports over specified time periods to generate incidence estimates. This readily available data can be used to inform the deployment of limited control interventions, thus maximise their impacts. However, case reporting is heavily influenced by disease awareness and variable healthcare infrastructure, which can distort generated measures [5]. Alternatively, population-based surveys or cohort studies including seroprevalence [6] and entomological surveys [7] can provide more reliable estimates of disease risk within their operational setting and are considered the gold standards for measuring long-term average transmission intensity. Yet, surveys are labour-intensive and difficult to conduct routinely over large geographical areas, so have limited use for routine surveillance proposes.

Recently, age-stratified incidence measures of case reports have been proposed as more suitable indicators of transmission intensity [5, 8, 9]. In areas of high transmission, the burden of disease is believed to disproportionately impact younger individuals compared to lower transmission areas where individuals likely report with dengue later in life due to the accumulation of immunity over time. This approach helps counter bias introduced by variable health care infrastructures and disease awareness although suffers two major caveats. First, case reporting is dependent on a variety of different diagnostics between and within endemic countries. Surveillance operations rely on non-specific diagnostics or clinical manifestations for notifying dengue [10]. Consequently, other co-endemic febrile infections, which manifest similarly to dengue and are common among children may prompt more younger individuals to seek care and be misdiagnosed as dengue infections [11, 12]. Second, among those reporting with true dengue infections, routinely used diagnostics are currently unable to distinguish those experiencing primary or post-primary infections [1]. Therefore, the age of case reports may be influenced by spatio-temporal imbalances in reported dengue immune status. Stratifying age estimates among those truly experiencing their primary infection may help to overcome these limitations.

In the Philippines, dengue has become a major contributor to mortality and morbidity over the past 50 years with all four serotypes believed to be endemic across urban centres [13]. Existing dengue surveillance operations consist of collating all reported dengue infections and surveying a subset for subsequent laboratory analysis. The serum is collected from sampled case reports for molecular and serological testing to allow spatio-temporal monitoring of dengue serotypes. Prior to this study, we utilised laboratory data from surveyed dengue patients to develop an algorithm that accurately determines individual primary or post-primary (secondary, tertiary or quaternary) immune status [14]. Despite this, it remains unknown how surveillance metrics derived from this algorithm can best be used to routinely characterise the FOI. Previous studies have demonstrated that the FOI can be reasonably estimated from age-stratified incidence rates [5, 9]. However, it remains unknown if other, easily computed, surveillance metrics represent surrogate indicators of the FOI. Here, we investigated whether laboratory/non-laboratory surveillance metrics correlate with the FOI according to age-seroprevalence and be used to routinely predict the burden of dengue across urban centres.

## Methods

### Data collection

Dengue is a notifiable disease among all disease reporting units (DRUs) across the Philippines and is recorded in line with The Philippine Integrated Disease Surveillance and Response (PIDSR) manual and WHO criteria [15]. Basic epidemiological data are collected from suspected dengue patients, including age, sex, date of symptom onset, date of reporting, patient/DRU address (barangay, municipality, province, region), symptoms and outcome. According to WHO criteria, dengue symptoms are classified among patients as those with acute febrile illness coupled with either no warning signs of dengue (abdominal pain, persistent vomiting, fluid accumulation, mucosal bleeding, lethargy and/or live enlargement) or severe dengue (severe plasma leakage, bleeding and/or organ impairment). All case reports were collated by the Philippine Epidemiological Bureau (Department of Health).

In addition to collated case reports, established laboratory surveillance operations performed by the Research Institute for Tropical Medicine (RITM—the research arm of the Department of Health) orchestrate annual cross-sectional surveys of dengue case reports across the country for further laboratory analysis. Participating DRUs, both sentinel and non-sentinel, collect single serum samples and basic epidemiological information (according to PIDSR criteria) from consenting, reporting dengue patients. Sentinel DRUs, including major hospitals, randomly select five case report samples per week. Non-sentinel DRUs comprising of any health facility across the Philippines that experiences a marked increase in suspected dengue case reporting according to PIDSR criteria also collected samples from case reports. For the purposes of this study, surveyed case report data were provided between 2014 and 2018 (*N* 20,666).

**Fig. 1:**
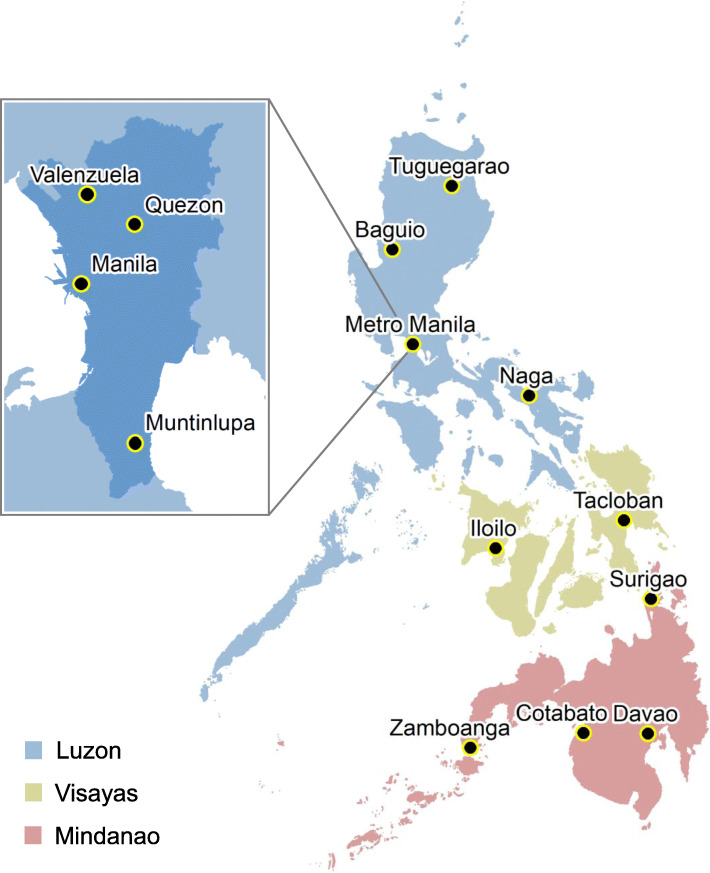
Location of 13 study-participating cities across the three principle geographical divisions of the Philippines that routinely provided serum samples between 2014 and 2018

During the study period, a total of 13 cities across the Philippines routinely provided serum samples from dengue patients who reported and resided in the same city (*N* 11,906) (Fig. 1) (Additional file 1). In these same cities between 2014 and 2017, a total of 80,043 case reports reported and lived in these corresponding cities. The population age structures of these cities according to the Philippine 2015 census (Philippine Statistics Authority) are shown in Additional file 2.

### Laboratory procedures

Serum samples were stored at −80°C at the RITM before molecular and serological assay. Serum samples were assayed using a serotype-specific, fourplex real-time reverse transcriptase PCR nucleic acid detection assay as described in [16]. Briefly, serotype-specific RNA is detected and amplified yielding a critical threshold (Ct) value that categorises samples as DENV1/2/3 or 4 positive (Ct<36) or DENV negative (Ct>36). In addition, samples were assayed for anti-DENV IgM and IgG using Panbio® capture ELISA kits (Cat No: 01PE10/01PE20, Alere, Brisbane, Australia) in accordance with the manufacturer’s instructions. ELISA plates contain antigen that captures host anti-DENV NS1 antibodies specific to all four serotypes and include calibrators that normalise optical density outputs to yield IgM and IgG panbio units.

### Data analysis

We categorised the dengue immune status (primary, post-primary, historical and negative) of the surveyed cases reports, using laboratory data, according to a previously developed algorithm [14]. Prior to categorisation, we generated additional variables: IgG to IgM ratio (IgG panbio units/IgM panbio units) and disease day (symptom onset date—reporting date). We excluded those under 6 months of age (0.4% 91/20,666) due to the potential influence of maternal antibodies and individuals who reported ≥6 days after symptom onset due to the algorithm’s inability to categorise convalescent infections. Details of the immune status algorithm are described in Additional file 3. Briefly, suspected dengue patients were categorised as active (PCR+ or IgM+) or non-active (PCR- and IgM-) dengue infections. Active dengue infections were further categorised as primary or post-primary (dengue infection with at least one previous flavivirus infection). Non-active dengue was classified as negative (anti-DENV IgG-) or historical (anti-DENV IgG+) for dengue.

Across the Philippines, we investigated temporal patterns in reported dengue immune status and serotype by stratifying immune status (primary and post-primary) among active dengue infections and dengue serotype (DENV1-4) among those DENV PCR+ over 30-day intervals. Among those reporting with primary and post-primary infections, we explored age-stratified disease severity patterns. Univariable logistic regression models were used to calculate odds ratios of being severe opposed to non-severe (warning signs and no warning signs) with the explanatory variables being age-stratified primary or post-primary dengue immune status. Regression models were fit using the ‘Logit’ command in STATA (v.16).

In each of the 13 sampled cities, we estimated all age and age-stratified (under 5 years and 10 years) aggregated annual dengue incidence rates among those who reported and resided in the same city. To estimate the average city population at risk of infection, we utilised city-specific population data from the 2015 population census (Philippine Statistics Authority) and population growth rates calculated between 2010 and 2015 (Additional file 2). City-specific population growth rates were used to estimate the population in each of the non-surveyed years. Average incidence per annum per 1000 persons equated to the number of case reports over the persons years at risk (average population over the study period multiplied by 4 years) multiplied by 1000 (Additional file 4).

In sampled cities, we estimated the average annual FOI estimated over the study period among non-active dengue infections (IgG+ historical and IgG-negative dengue cases). Seroprevalence corresponded to the proportion of the non-active dengue cases who were IgG seropositive to any serotype. Catalytic models were fit, by maximum likelihood, to estimate age-seroprevalence and derive seroconversion rates which correspond to the average annual rate individuals seroconvert from IgG− to IgG+ status; a rate analogous to the FOI. Under the assumption, individuals remain IgG+ after seroconversion and all circulating serotypes contributed equally to transmission, a simple catalytic model (Eq. 1) calculates the probability of being IgG seropositive at specific ages (*a*) by fitting a constant force of infection parameter (***λ***) by least squares:

1 $$ P(a)=\left[1-{\mathrm{e}}^{-\uplambda \mathrm{a}}\right] $$

Assuming IgG antibodies can wane over time to low levels undetectable according to the commercial ELISA kits, as previously shown in [17–19], a reversible catalytic model (Eq. 2) additionally estimates a seroreversion rate (the average annual rate individuals serorevert back to IgG-status) by fitting an additional seroreversion parameter (*ρ*), by least squares:

2 $$ P(a)=\frac{\lambda }{\lambda +\rho}\left[1-{e}^{-\left(\lambda +\rho \right)a}\right] $$

To determine which catalytic model, simple or reversible, was most appropriate to estimate the FOI, we used AIC (Akaike information criterion) to determine superior model fits in each city. Catalytic models were fitted using a rho-constrained/unconstrainted ‘revcat’ command in STATA (v.16).

We investigated whether routinely collected surveillance metrics represent surrogate indicators of the FOI at the city level, we explored their statistical association with the city FOI aggregated over the study period. Non-laboratory surveillance metrics investigated included all age/under 5/under 10 average annual dengue incidence, mean age of all reported case reports/case reports with dengue warning signs/case reports with severe dengue. Laboratory-derived surveillance metrics included the mean age of laboratory-confirmed active dengue infections/primary dengue infections/post-primary dengue infections. The strength of association between the city-aggregated FOI and averaged surveillance metrics were assessed using Pearson’s correlation coefficient (*ρ*, *p* value). Among surveillance metrics that significantly correlated with the FOI at the city level (*ρ*, *p* value<0.05), exponential regression models were used to generate predicted FOI estimates. Lrtests were adopted to justify exponential over linear model fits (*p* value<0.05). Models were fit using a non-linear ‘nl’ command in STATA (v.16). FOI estimates were subsequently converted to annual attack rates (AR) (Eq. 3) to determine the proportion of the sampled population who became exposed to dengue in each city per year:

3 $$ \mathrm{AR}=1-{\mathrm{e}}^{-\left(\uplambda \right)} $$

## Results

### Data description

Demographic characteristics of the sampled and collated acute case reports across the Philippines are highlighted in Table 1. Similar demographic patterns were observed among both case report datasets. The majority of patients were aged 6–15 years of age, reported 3–4 days after the onset of symptoms and presented with warning signs of dengue. Among the surveyed case reports with laboratory data, we revealed 18% (3310/18,366) were primary infections, 62.9% (11,560/18,366) were post-primary infections, 12.5% (2297/18,366) were historical dengue infections and 6.5% (1199/18,366) were negative dengue infections.

**Table 1:** Study population demographics. Characteristics of sampled and collated acute dengue case reports across the Philippines. Excludes patients who reported more than 5 days after symptom onset and those under the age of 6 months

Demographics	Surveyed case reports		Collated case reports	
	%*n*	*n*	%*n*	*n*
**Age (years)**				
<5	15.9	2920	17.1	80,046
6–15	45.6	8371	39.1	182,999
16–25	23.4	4299	24.8	116,062
26–40	9.3	1716	11.5	53,803
>41	5.8	1060	7.5	35,117
**Sex**				
Female	47	8624	47.3	221,446
Male	53	9742	52.7	246,581
**Disease day**				
<2	28.5	5233	31.8	148,933
3–4	58	10,652	54.6	255,352
5	13.5	2481	13.6	63,742
**Symptoms**				
No warning signs	14.2	2617	16.5	77,006
Warning signs	53.4	9811	50.3	235,619
Severe dengue	5.1	929	4.0	18,506
Non-disclosed	27.3	5009	29.2	136,896
**DENV immune status**				
Primary	18	3310	–	–
Post-primary	62.9	11,560	–	–
Historical	12.5	2297	–	–
Negative	6.5	1199	–	–
**Total**	100.0	18,366	100.0	468,027

**Fig. 2:**
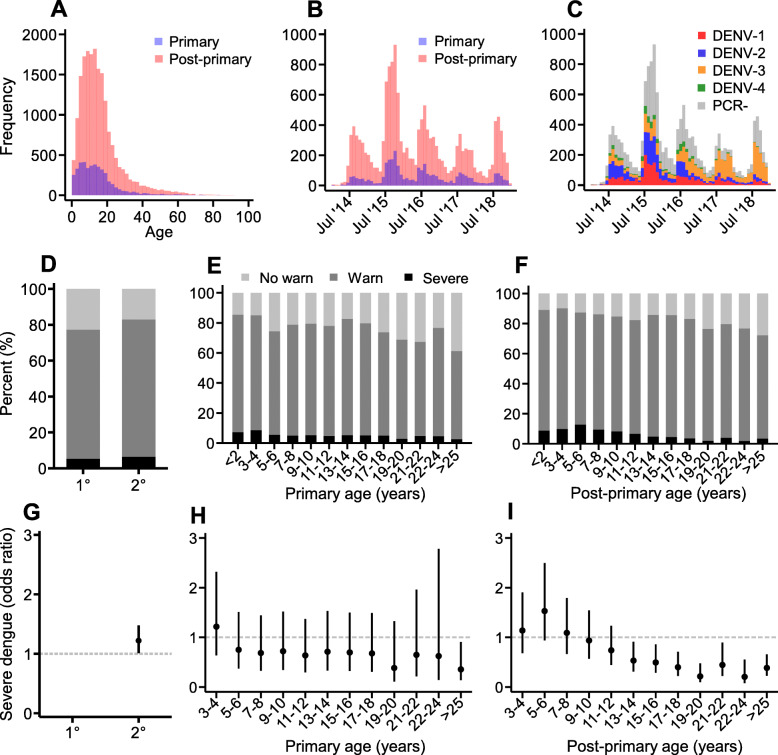
Immuno-epidemiological characteristics of dengue infections reporting across the Philippines between 2014 and 2018. **A** Age distribution of the primary and post-primary dengue infections. **B** Temporal patterns in reporting primary and post-primary dengue infections. **C** Temporal patterns in reporting DENV serotypes among active dengue infections. **D** Clinical manifestations of the primary and post-primary dengue infections. **E** Age-stratified clinical manifestations of primary dengue infections. **F** Age-stratified clinical manifestations of post-primary dengue infections. **G** Odds ratio [95% CI] for post-primary dengue infections to present with severe disease compared to primary dengue infections. **H** Odds ratios [95% CI] for primary dengue infections to present with severe disease by age in years compared to those under 2 years. **I** Odds ratios [95% CI] for post-primary dengue infections to present with severe disease by age in years compared to those under 2 years. 1° primary dengue infections. 2° Post-primary dengue infections

Overall dengue immuno-epidemiological patterns between 2014 and 2018 revealed both primary and post-primary dengue infections were mainly children and young adults (Fig. 2A) and continuously reported to health facilities during the study period (Fig. 2B). Among PCR+ case reports, we revealed by 2017 and 2018, DENV-3 replaced DENV-1/2 as the most dominant serotype across the Philippines (Fig. 2C). Among those with disclosed symptom data (72.7% 13,357/18366), we observed similar clinical manifestations among primary and post-primary infections (Fig. 2D), whereby younger individuals tended to present with more dengue warning signs or severe dengue (Fig. 2E & F). Despite this, post-primary infections were slightly more likely to present with severe disease compared to primary infections (OR 1.22 [95%CI 1.01–1.48] *p* value 0.039) (Fig. 2G). Among young primary infections, we observed no age-stratified severity trends: individuals over 2 years, yet younger than 25 years, were statistically no more/less likely to present with severe disease than those under 2 years (Fig. 2H). In contrast, young children with post-primary infections were more likely to present with severe disease compared to their elders. Compared to post-primary infections under 2 years, those over 12 years were significantly less likely to present with severe disease (Fig. 2I).

### City-level dengue transmission dynamics

Among 13 study-participating cities during the study period, we observed variation in estimated crude and age-stratified incidence rates. All-age incidence was highest in Baguio (3.75 cases per annum per 1000 [95%CI 3.65–3.86]) and lowest in Valenzuela (0.49 cases per annum per 1000 [95%CI 0.46–0.52]). After stratifying among younger age groups, city incidence rates changed. The highest incidence rate among those under ten was observed in Surigao (7.17 cases per annum per 1000 [95%CI 6.71–7.65]) while the lowest rate was again in Valenzuela (0.84 cases per annum per 1000 [95%CI 0.76–0.93]) (Additional file 4).

**Fig. 3:**
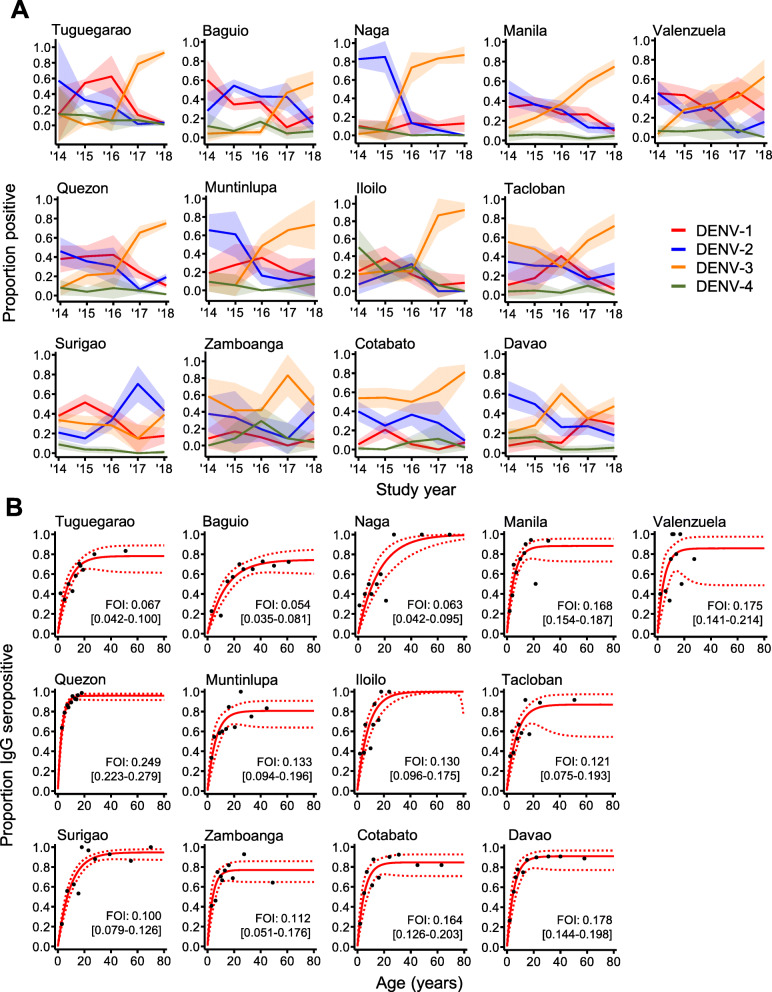
City-level immuno-epidemiological characteristics of the reporting dengue population between 2014 and 2018. **A** The percentage of PCR-positive patients who are DENV-1/2/3/4-positive. Shading: 95% CIs. **B** The dengue FOI in 13 cities across the Philippines aggregated between 2014 and 2018. FOI corresponds to the average annual seroconversion rate calculated among non-active dengue cases (historical and negative dengue cases). Black dots: observed age-seroprevalence. Red lines: fitted age-seroprevalence with 95%CIs (red dash)

Across cities, we revealed prominent spatio-temporal heterogeneity in serotype dominance (Fig. 3A) and immune status reporting (primary/post-primary) (Additional file 5). During 2015/16, cities in northern Luzon were mainly burdened by DENV-1/2, yet by 2017 and 2018, DENV-3 had become the most dominant. In contrast, cities in Mindanao were burdened with DENV-3 throughout the entire study period, and in Surigao, DENV-2 replaced DENV-3 as the most dominant serotype in 2016. It should be noted that for nearly every year among all sampled cities, all four serotypes of dengue were detected among reporting PCR+ patients. Regarding the immune status of the population, patients in the cities of southern Mindanao (Zamboanga, Cotabato and Davao) had a higher probability of reporting as post-primary opposed to primary dengue infections compared to those in cities of Luzon. Within cities, we observed temporal variation in immune status reporting. For instance, in Valenzuela (Metro Manila), primary dengue reporting significantly increased during 2016, while in Tuguegarao, fell between 2015 and 2018.

Furthermore, we estimated the average annual dengue FOI among the 13 cities aggregated over the study period. In all cities, the reversible, opposed to the simple, catalytic model produced superior model fits (reversible model AIC < simple model AIC) (Additional file 6). We therefore opted to estimate the FOI (or SCR) in each city using the more complex, reversible model. Between each city, the FOI rate ranged from 0.054 [95%CI 0.036-0.081] per year in Baguio city to 0.249 [95%CI 0.223–0.279] per year in Quezon city demonstrating spatial heterogeneity in the transmission intensity (Fig. 3B).

### Estimating the city-level FOI from routinely collected surveillance metrics

**Fig. 4:**
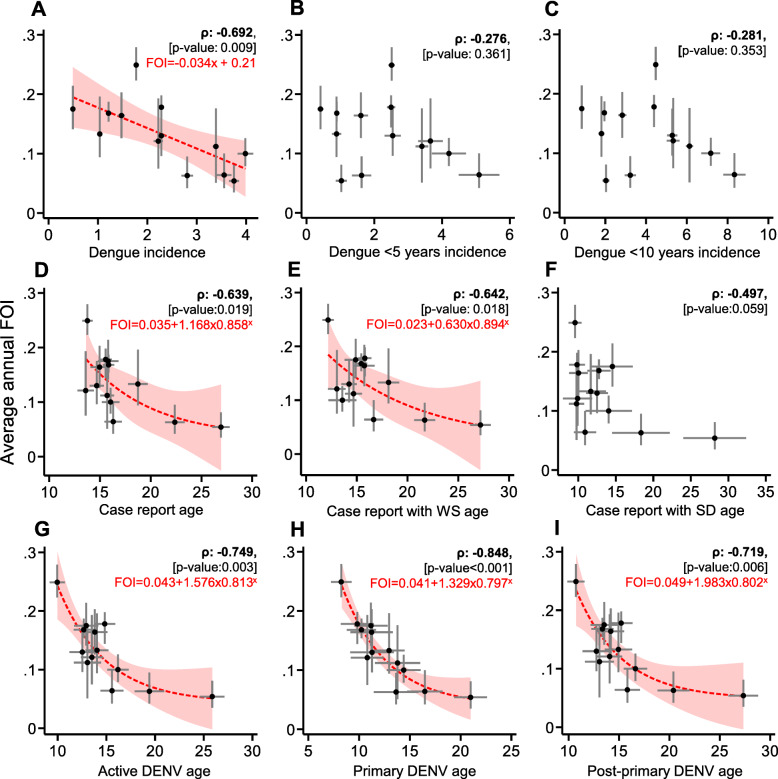
The city and study period aggregated association between the average annual FOI and surveillance metrics. **A** Crude incidence. **B** Under five incidence. **C** Under 10 incidence. **D** Mean age of case reports. **E** Mean age of case reports with warning signs. **F** Mean age of case reports with severe dengue. **G** Mean age of active infections. **H** Mean age of primary dengue infections. **I** Mean age of post-primary dengue infections. *ρ* Pearson’s R. **A**–**F** Data from passive surveillance. **G**–**I** Data from laboratory surveillance. Red dash: predicted FOI according to regression models for metrics with statistically significant associations with FOI (***ρ***, *p* value>0.05). Linear regression models were favoured for crude incidence while exponential regression models had superior model fits for mean case report age, case report with warning signs age, active DENV age, primary DENV age and post-primary DENV age (Lrtest, *p* value<0.05). *X* refers to the annual mean surveillance metric

To assess whether data from routine dengue case surveillance activities could be used to estimate the FOI in cities, we explored statistical associations between catalytic model estimated city-level FOI against age/age-stratified incidence and the mean age of different types of dengue infections (Fig. 4). We observed a negative correlation between the FOI and all-age average annual incidence, whereby incidence increased with decreasing FOI (*ρ* −0.692, *p* value 0.009). No association was identified between the city-level FOI and average annual age-stratified (under 5 and under 10 years) incidence rates (*ρ*, *p* value>0.05). Among all those who reported suspected dengue, we identified a negative correlation between the FOI and the mean age of all case reports (*ρ* −0.639, *p* value 0.019). For those who reported with dengue warning signs, we identified a similar association where the decreasing FOI correlated with the increasing mean age (*ρ* −0.642, *p* value: 0.018). In contrast, we observed a weak association between the FOI and the mean age of severe case reports (*ρ* −0.497, *p* value 0.059). Among those with laboratory-confirmed dengue infections, we identified stronger associations between mean age and the FOI. The mean age of active dengue infections (primary and post-primary) increased with decreasing FOI (*ρ*:–0.749, *p* value 0.003). An association that was strengthened after stratifying among only primary infections (*ρ* −0.848, *p* value<0.001), yet remained similar when stratifying by post-primary infections (*ρ* −0.719, *p* value 0.006). We repeated these associations using FOI estimates generated from simple, opposed to reversible, catalytic models and still found the mean age of primary infections had the strongest association with the FOI (*ρ* −0.720, *p* value 0.005) (Additional file 7).

For each surveillance metric that demonstrated a statistically significant association with the FOI, we estimated the predicted FOI using exponential regression models where appropriate (*ρ*, *p* value<0.05). For each statistically associated metric, except incidence, exponential opposed to linear model fits were favoured (Lrtest, *p* value<0.05). Using these regression models, we estimated annual dengue attack rates from predicted FOI estimates from the most associated laboratory- and non-laboratory-derived metrics: the mean age of laboratory-confirmed primary dengue infections (Table 2) (Additional file 8) and the mean age of case reports with warning signs (Additional file 9). Annual trends in the mean primary age, and thus predicted attacks rates, revealed spatio-temporal heterogeneity in dengue transmission intensity among sampled cities. In Zamboanga city, the attack rate (AR) increased dramatically from 4% [95%CI 4–4%] of the population being exposed in 2014 to 30% [95%CI 19–46%] in 2018. In Manila city, the AR decreased during the study period from 27% [95%CI 20–36%] in 2015 to 10% [95%CI 8–14%] in 2018. In Quezon city, the AR remained high between 17% and 23% during the entire study period which is consistent with the estimated FOI according to the catalytic model (AR 22% [95%CI 20–24%]). For some cities (Davao, Naga), a change in serotype dominance corresponded with increase in AR; however, this was not observed in others (Tuguegarao, Manila). We also predicted the annual ARs according to the mean age of case reports with warning signs that also highlighted spatio-temporal heterogeneity in FOI, yet was less consistent with the overall AR according to the catalytic model (Additional file 10).

**Table 2:**
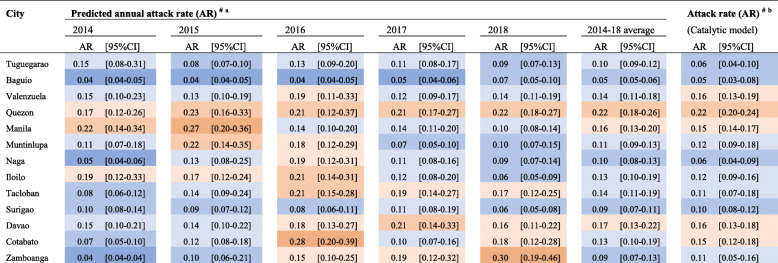
The annual predicted dengue attack rates among sampled cities between 2014 and 2018 according to the mean age of laboratory confirmed primary dengue infections. 95%CIs correspond to the upper and lower 95%CIs of the annual mean age of primary infections. Attack rates according to catalytic models were derived from FOI estimates from reversible catalytic models

^#^Attack rate (AR): 1-exp^(-FOI)^

^a^Estimated annual attack rate according to the FOI derived from the mean primary DENV age (FOI=0.041+1.329×0.797×), where *x* equals the annual mean primary dengue age

^b^Estimated annual attack rate according to the FOI derived from the reversible catalytic model, Eq. 2, where FOI equals *λ*

Attack rate

0.25-0.30 0.20-0.25 0.15-0.20 0.10-0.15 0.05-0.10 0.05-0.10

## Discussion

We explored methods to routinely describe dengue transmission intensity among cities across the Philippines. Using data from surveillance operations, we found the mean age of laboratory-confirmed primary dengue infections and the mean age of case reports with warnings signs, to a lesser extent, represented suitable surrogate indicators of the FOI. Both these easily computed metrics correlated better than crude and age-stratified incidence with the FOI estimated from seroprevalence data. Our results highlight prominent spatio-temporal heterogeneity in dengue serotype dominance and reported immune status and the FOI across urban centres in the Philippines.

It is well documented that secondary, opposed to primary, dengue infections are at a greater risk of developing severe disease [20–22], yet we found those categorised as post-primary infections were only slightly more likely to present with severe disease than primary infections. This made us suspect that some of those classified as post-primary were tertiary and quaternary dengue infections, given these types of infections are thought to be less severe than secondary infections, although it is still widely assumed post-secondary infections are often asymptomatic [2, 23]. Furthermore, we identified all four serotypes of dengue in every city almost every year making it plausible for individuals residing in urban centres to suffer multiple dengue infections over their lifetime. Lastly, we found that younger, opposed to older, post-primary dengue infections were at greater risk of presenting with severe disease. A trend possibly attributed to younger and older post-primary infections representing secondary and post-secondary infections, respectively, as shown in [2], although this may also be attributable to a different disease presentation in older people.

Our city FOI estimates, aggregated over 5 years and generated from catalytic models, were comparable to a 2016 estimate in Cebu (central Philippines) [23] and revealed a long-term spatial heterogeneity in dengue transmission intensity, similarly shown in China [24] and Colombia [25]. We found catalytic models fit with sero-reversion parameters resulted in superior model fits despite sero-reversion being very low across sampled cities. This is consistent with previous findings that documented a minority of dengue cases, who were likely exposed once, experienced IgG waning [17, 18]. To determine whether the FOI could be estimated from data routinely collected by dengue surveillance operations, we explored the association between FOI, estimated from catalytic models, against various dengue surveillance metrics. Surprisingly, aggregated at the city level and over the study period, all-age reported incidence was higher in lower FOI areas. This trend however has previously been reported in Singapore [26] and was thought to be attributed to an age shift in disease burden. In low transmission areas, individuals are more likely to experience a dengue infection at a later stage in their life when they may have heightened individual disease awareness and possibly easier access to care. Consequently, low transmission areas may experience increased case reporting. In addition, this trend could also be explained by reduced health-seeking behaviour in high FOI cities wherein individuals refrain from reporting with symptoms as they are aware of many others with this often mild, self-limiting disease. In contrast in low FOI areas, individuals may be more prompted to seek care as they less familiar with the disease and are more concerned by unfamiliar symptoms. However, future population KAP (knowledge attitudes and practises) surveys in high- and low-transmission settings would be valuable in better describing these health-seeking patterns. Finally, this disparity could partially be explained by the FOI and incidence being measures of infection and disease, respectively. FOI estimates also include those asymptomatic with dengue who are thought to account for approximately 75% of all dengue cases and would unlikely seek treatment [27]. More surprisingly however, after stratifying incidence by age, we observed no association with the FOI which contradicts findings in [9]. We speculate that variable healthcare access and reporting across the country may account for this irregularity and remains an area of continued investigation. Together, these findings suggest that simply allocating control interventions to areas with elevated reported incidence might exclude regions with higher burdens of infection.

In cities, we found that the average age of reported dengue infections with clinical dengue outcomes increased with decreasing aggregated transmission intensity. Interestingly, the mean age of those reporting with just warning signs of dengue, opposed to severe dengue, proved a superior indicator of FOI. The poor specificity dengue clinical diagnosis is well documented as other febrile infections can present with similar disease manifestations [28–30]. Yet, compared to warning signs, severe disease is a far rarer outcome, particularly among primary and older post-primary infections. Moreover, in Thailand, reported severe dengue manifestations have been shown to be highly spatially and temporally heterogenous due to climactic factors [31]. Together, these factors likely account for the lack of association between FOI and age of case reports with severe disease.

In contrast to the age of clinically diagnosed dengue patients, the age of laboratory-confirmed dengue cases had a stronger association with the FOI, likely a consequence of excluding other co-endemic febrile infections. Yet, among laboratory confirmed infections, the age of those reporting with primary, rather than post-primary, infections proved a better indicator of FOI. We propose two main factors account for this difference. Age patterns of reporting post-primary infections might be influenced by spatio-temporal imbalances in immune status reporting, observed in this study and previously in Vietnam [32] and Taiwan [33]. In high transmission settings, where a higher proportion of the post-primary reporting population include tertiary/quaternary infections, the mean age would be higher than an equally high transmission setting where most of the post-primary infections were secondary infections. In contrast, the age of reported primary infections remains unaffected by imbalances in immune status reporting. Secondly, studies have shown that Zika, a structurally homologous virus to dengue, elicits cross-reactive IgG that primes individuals for secondary dengue infections [34, 35]. Therefore, spatio-temporal imbalances in potential Zika transmission across the Philippines may similarly impact the age of post-primary, yet not primary, dengue infection reporting. These interactions led us to propose the age at which individuals report with their first dengue infection is the better surrogate of transmission intensity which we used to estimate annual FOI across cities. It should be noted however, given that individuals are classified as primary or post-primary according to antibody titre and ratio thresholds, a minority of the study population with metrics on the cusp of these cut offs may have been misclassified [14].

Upon estimating city level, annual dengue attack rates according to the age of primary dengue infections, we demonstrated that yearly estimates correlated with overall attack rates according to the catalytic model. Patterns in predicted annual attack rates across cities revealed changing patterns in transmission intensity over time, as seen in Singapore [26]. We identified cities where transmission is stable, emerging and decreasing. Information that is critical for national dengue surveillance operations to plan for the future [36]. In Zamboanga, despite a relatively low overall FOI, annual predicted attack rates revealed that transmission intensity increased during the study period, a trend undetected in the collated incidence data. Zamboanga could therefore be earmarked for heightened surveillance activities to better characterise worrying predicted trends in the burden of infection. In Naga, we identified a jump in FOI coinciding with a switch in serotype dominance, yet this was unobserved in other cities. Approximating transmission intensity from the age of reported primary dengue infections has two major benefits. Firstly, existing surveillance operations can generate attack rates quickly and simply. Moreover, as a consequence of the exponential relationship, slight changes in the age of reported primary infections correspond to larger changes in transmission intensity. This enables FOI estimates to be generated at more granular temporal scales, which are more informative than long-term estimates derived from catalytic models. As well as describing dengue transmission intensity patterns, we speculate monitoring age patterns of reported primary cases could assist in dengue vaccine deployment. Currently, the only licenced dengue vaccine, Dengvaxia®, is recommended for individuals aged between 9 and 45 years in endemic areas, presumably as these likely include those with prior dengue exposure [37]. Our results highlight the spatio-temporal heterogeneity in the age patterns of those reporting with their first infection; therefore, monitoring the age of these infections could potentially help identify suitable age ranges in specific locations to receive vaccination, although individual pre-vaccination testing would still be necessary and this requires legislative changes.

There are limitations associated with these findings. FOI estimates were generated from reporting, non-active, dengue infections with/without previous dengue IgG exposure. Consequently, we may have oversampled individuals who were more prompted to seek health care if they experienced a prior dengue infection. Yet, only 25% of dengue infections are thought to experience symptoms that would prompt them to seek care [27], and we concluded this health-seeking bias was minimal. Secondly, our fitted catalytic model assumes serotypes contributed equally to transmission over time. Still, given we identified every serotype in each city and that serotype dominancy changed rapidly over the 5-year sampling period, it is reasonable to assume that over the previous decades, from which the FOI is estimated, periodic serotype dominancy may have resulted in serotypes contributing equally to overall transmission. Lastly, our analysis confirms the age of primary dengue infections is associated with FOI at the city level, yet it remains unconfirmed as to whether the age of reported dengue cases accurately represent transmission intensity at lower administrative levels where patients are more likely to cross administrative borders for care. Similarly, our analysis focused on revealing annual trends in dengue transmission intensity. It remains unknown whether seasonal biases may influence the reporting age of primary dengue infections at more granular temporal scales.

## Conclusion

We described methods for estimating city-level dengue transmission intensity using metrics obtained from both national laboratory and non-laboratory dengue surveillance operations. We revealed the mean age of those reporting with primary dengue infections correlated best with the FOI using laboratory data, while the mean age of those reporting with clinically diagnosed warning signs represented the best surrogate of FOI using non-laboratory surveillance data. Our work highlights the importance of laboratory dengue surveillance operations and provides a framework for other dengue-endemic countries to better characterise dengue transmission patterns and combat the global threat of this disease.

## Supplementary Information

**Additional file 1.** Serum samples collected from dengue case reports. The number of serum samples collected from surveyed dengue case reports who reported and resided in 13 cities across the Philippines between 2014 & 2018.

**Additional file 2.** Population demographics of study-participating cities.

**Additional file 3.** Methods used to determine primary and post-primary dengue immune status.

**Additional file 4.** City-aggregated dengue incidence estimates.

**Additional file 5.** Reported dengue immune status by year and city. The reported primary/post-primary immune status of reporting active dengue infection by year and city across the Philippines between 2014 and 2018. Vertical bars: 95%CI.

**Additional file 6.** FOI catalytic model comparison. Catalytic model fit comparison of simple versus reversible catalytic model used to estimate FOI among sampled cities. AIC: Akaike information criterion. Lower AIC (bold) indicates superior model fit.

**Additional file 7.** The city and study period aggregated association between the average annual FOI, according to simple catalytic models, and surveillance metrics. A: crude incidence. B: Under five incidence. C: Under 10 incidence. D: Mean age of case reports. E: Mean age of case reports with warning signs. F: Mean age of case reports with severe dengue. G: Mean age of active infections. H: Mean age of primary dengue infections. I: Mean age of post-primary dengue infections. ρ: Pearson’s R. A-F: Data from passive surveillance G-I: Data from laboratory surveillance. Red dash: predicted FOI according to regression models for metrics with statistically significant associations with FOI (ρ, p-value>0.05).

**Additional file 8.** Mean annual primary dengue age by city. The average annual age of reported primary dengue infections among study-participating cities between 2014 and 2018.

**Additional file 9.** Mean annual age of cases with dengue warning signs by city. The average annual age of reported dengue cases with warning signs among study-participating cities between 2014 and 2018.

**Additional file 10.** Annual city Attack rates by city according to the mean age of suspected dengue cases with warning signs.

## Data Availability

The datasets used in this study are available from the corresponding author on reasonable request.

## Abbreviations

AIC: Akaike information criterion
CI: Confidence interval
Ct: Critical threshold
DENV: Dengue virus
DOH: Department of Health
DRU: Disease reporting unit
ELISA: Enzyme-linked immunosorbent assay
FOI: Force of infection
IgG: Immunoglobulin G
IgM: Immunoglobulin M
OR: Odds ratio
PCR: Polymerase chain reaction
PIDSR: Philippine Integrated Disease Surveillance and Response
SCR: Sero-conversion rate
SRR: Sero-reversion rate
WHO: World Health Organization
ZIKV: Zika virus

## References

[1] Guzman MG, Gubler DJ, Izquierdo A, Martinez E, Halstead SB (2016). Dengue infection. Nat Rev Dis Prim.

[2] Wikramaratna PS, Simmons CP, Gupta S, Recker M. The effects of tertiary and quaternary infections on the epidemiology of dengue. Schneider BS. PLoS One. 2010; 5(8):e12347. PMID: 20808806 10.1371/journal.pone.0012347 PMC2925950 20808806

[3] Halstead SB. Dengue antibody-dependent enhancement: knowns and unknowns. In: Antibodies for Infectious Diseases: American Society of Microbiology; 2015. p. 249–71. PMID: 26104444. 10.1128/microbiolspec.AID-0022-2014 26104444

[4] Bowman LR, Donegan S, McCall PJ. Is dengue vector control deficient in effectiveness or evidence?: systematic review and meta-analysis. James AA, editor. PLoS Negl Trop Dis. 2016; 10(3):e0004551. PMID: 26986468 10.1371/journal.pntd.0004551 PMC4795802 26986468

[5] Rodriguez-Barraquer I, Salje H, Cummings DA. Opportunities for improved surveillance and control of dengue from age-specific case data. Elife. 2019;8. PMID: 31120419. 10.7554/eLife.45474. 10.7554/eLife.45474 PMC6579519 31120419

[6] WHO (2017). Informing vaccination programs: a guide to the design and conduct of dengue serosurveys.

[7] Basker P, Kannan P, Porkaipandian RT, Saravanan S, Sridharan S, Kadhiresan M (2013). Study on entomological surveillance and its significance during a dengue outbreak in the District of Tirunelveli in Tamil Nadu, India. Osong Public Heal Res Perspect.

[8] O’Driscoll M, Imai N, Ferguson NM, Hadinegoro SR, Satari HI, Tam CC, et al. Spatiotemporal variability in dengue transmission intensity in Jakarta, Indonesia. Azman AS, editor. PLoS Negl Trop Dis. 2020; 14(3):e0008102. PMID: 32142516 10.1371/journal.pntd.0008102 PMC7080271 32142516

[9] Imai N, Dorigatti I, Cauchemez S, Ferguson NM. Estimating dengue transmission intensity from case-notification data from multiple countries. Althouse B, editor. PLoS Negl Trop Dis. 2016; 10(7):e0004833. PMID: 27399793 10.1371/journal.pntd.0004833 PMC4939939 27399793

[10] Raafat N, Blacksell SD, Maude RJ (2019). A review of dengue diagnostics and implications for surveillance and control. Trans R Soc Trop Med Hyg.

[11] Salje H, Cauchemez S, Alera MT, Rodriguez-Barraquer I, Thaisomboonsuk B, Srikiatkhachorn A, Lago CB, Villa D, Klungthong C, Tac-An IA, Fernandez S, Velasco JM, Roque VG, Nisalak A, Macareo LR, Levy JW, Cummings D, Yoon IK (2016). Reconstruction of 60 years of chikungunya epidemiology in the philippines demonstrates episodic and focal transmission. J Infect Dis.

[12] Lopez AL, Raguindin PF, Aldaba JG, Avelino F, Sy AK, Heffelfinger JD, Silva MWT (2021). Epidemiology of Japanese encephalitis in the Philippines prior to routine immunization. Int J Infect Dis.

[13] World Health Organisation (WHO) (2017). Western pacific regional action plan for dengue prevention and control (2016).

[14] Biggs JR, Sy AK, Brady OJ, Kucharski AJ, Funk S, Reyes MAJ, Quinones MA, Jones-Warner W, Tu YH, Avelino FL, Sucaldito NL, Mai HK, Lien LT, Do Thai H, Nguyen HAT, Anh DD, Iwasaki C, Kitamura N, Yoshida LM, Tandoc AO, la Paz ECD, Capeding MRZ, Padilla CD, Hafalla JCR, Hibberd ML (2020). A serological framework to investigate acute primary and post-primary dengue cases reporting across the Philippines. BMC Med.

[15] Department of Health (DoH). Philippine integrated disease surveillance and response: National Epidemiology Centre; 2014.

[16] Johnson BW, Russell BJ, Lanciotti RS (2005). Serotype-specific detection of dengue viruses in a fourplex real-time reverse transcriptase PCR assay. J Clin Microbiol.

[17] Luo S, Cui W, Li C, Ling F, Fu T, Liu Q, Ren J, Sun J (2018). Seroprevalence of dengue IgG antibodies in symptomatic and asymptomatic individuals three years after an outbreak in Zhejiang Province, China. BMC Infect Dis.

[18] Shah PS, Alagarasu K, Karad S, Deoshatwar A, Jadhav SM, Raut T, Singh A, Dayaraj C, Padbidri VS (2019). Seroprevalence and incidence of primary dengue infections among children in a rural region of Maharashtra, Western India. BMC Infect Dis.

[19] Imai N, Dorigatti I, Cauchemez S, Ferguson NM. Estimating dengue transmission intensity from sero-prevalence surveys in multiple countries. Hay SI, editor. PLoS Negl Trop Dis. 2015; 9(4):e0003719. 10.1371/journal.pntd.0003719 PMC4400108 25881272

[20] Guzman MG, Alvarez M, Halstead SB (2013). Secondary infection as a risk factor for dengue hemorrhagic fever/dengue shock syndrome: an historical perspective and role of antibody-dependent enhancement of infection. Arch Virol.

[21] Katzelnick LC, Gresh L, Halloran ME, Mercado JC, Kuan G, Gordon A (2017). Antibody-dependent enhancement of severe dengue disease in humans. Science.

[22] St John AL, Rathore APS (2019). Adaptive immune responses to primary and secondary dengue virus infections. Nat Rev Immunol.

[23] Alera MT, Srikiatkhachorn A, Velasco JM, Tac-An IA, Lago CB, Clapham HE, et al. Incidence of dengue virus infection in adults and children in a prospective longitudinal cohort in the Philippines. Carvalho MS, editor. PLoS Negl Trop Dis. 2016; 10(2):e0004337. PMID: 26845762 10.1371/journal.pntd.0004337 PMC4742283 26845762

[24] Cheng Q, Lu X, Wu JT, Liu Z, Huang J (2016). Analysis of heterogeneous dengue transmission in Guangdong in 2014 with multivariate time series model. Sci Rep.

[25] Estupiñán Cárdenas MI, Herrera VM, Miranda Montoya MC, Lozano Parra A, Zaraza Moncayo ZM, Flórez García JP, Rodríguez Barraquer I, Villar Centeno LÁ (2020). Heterogeneity of dengue transmission in an endemic area of Colombia. PLoS Negl Trop Dis.

[26] Tan LK, Low SL, Sun H, Shi Y, Liu L, Lam S, Tan HH, Ang LW, Wong WY, Chua R, Teo D, Ng LC, Cook AR (2019). Force of infection and true infection rate of dengue in singapore: implications for dengue control and management. Am J Epidemiol.

[27] Bhatt S, Gething PW, Brady OJ, Messina JP, Farlow AW, Moyes CL, Drake JM, Brownstein JS, Hoen AG, Sankoh O, Myers MF, George DB, Jaenisch T, Wint GRW, Simmons CP, Scott TW, Farrar JJ, Hay SI (2013). The global distribution and burden of dengue. Nature..

[28] Dussart P, Duong V, Bleakley K, Fortas C, Lorn Try P, Kim KS, Choeung R, in S, Andries AC, Cantaert T, Flamand M, Buchy P, Sakuntabhai A (2020). Comparison of dengue case classification schemes and evaluation of biological changes in different dengue clinical patterns in a longitudinal follow-up of hospitalized children in Cambodia. PLoS Negl Trop Dis.

[29] Cavailler P, Tarantola A, Leo YS, Lover AA, Rachline A, Duch M, Huy R, Quake AL, Kdan Y, Duong V, Brett JL, Buchy P (2016). Early diagnosis of dengue disease severity in a resource-limited Asian country. BMC Infect Dis.

[30] Low JGH, Ong A, Tan LK, Chaterji S, Chow A, Lim WY, Lee KW, Chua R, Chua CR, Tan SWS, Cheung YB, Hibberd ML, Vasudevan SG, Ng LC, Leo YS, Ooi EE (2011). The early clinical features of dengue in adults: challenges for early clinical diagnosis. PLoS Negl Trop Dis.

[31] Xu Z, Bambrick H, Yakob L, Devine G, Lu J, Frentiu FD, Yang W, Williams G, Hu W (2019). Spatiotemporal patterns and climatic drivers of severe dengue in Thailand. Sci Total Environ.

[32] Lam HM, Phuong HT, Thao Vy NH, Le Thanh NT, Dung PN, Ngoc Muon TT (2019). Serological inference of past primary and secondary dengue infection: implications for vaccination. J R Soc Interface.

[33] Lin C-C, Huang Y-H, Shu P-Y, Wu H-S, Lin Y-S, Yeh T-M (2010). Characteristic of dengue disease in Taiwan: 2002-2007. Am J Trop Med Hyg.

[34] Katzelnick LC, Narvaez C, Arguello S, Lopez Mercado B, Collado D, Ampie O (2020). Zika virus infection enhances future risk of severe dengue disease. Science (80- ).

[35] Martín-Acebes MA, Saiz J-C, Jiménez de Oya N. Antibody-dependent enhancement and zika: real threat or phantom menace? Front Cell Infect Microbiol. 2018;8 PMID: 29497604. 10.3389/fcimb.2018.00044 PMC5818409 29497604

[36] World Health Organization (WHO) (2009). Dengue: guidelines for diagnosis, treatment, prevention and control.

[37] Aguiar M, Stollenwerk N (2018). Dengvaxia: age as surrogate for serostatus. Lancet Infect Dis.

