# Supplementary material for: Estimating the annual dengue force of infection from the age of reporting primary infections across urban centres in endemic countries
Source: BMC Med. 2021 Sep 30;19:217. doi: 10.1186/s12916-021-02101-6 (PMC8482604; doi:10.1186/s12916-021-02101-6)
Supplement: Supplementary file 1 — Additional file 1. Serum samples collected from dengue case reports. The number of serum samples collected from surveyed dengue case reports who reported and resided in 13 cities across the Philippines between 2014 & 2018. [file 12916_2021_2101_MOESM1_ESM.pdf]

Island Region	City	DRU	Annual serum samples collected (n)					
			2014	2015	2016	2017	2018	Total
Luzon	Tuguegarao	Cagayan Valley Medical Center	15	612	25	88	488	1228
Luzon	Baguio	Baguio General Hospital	49	466	168	136	194	1013
		Pines City Doctors Hospital	1	2	127	0	0	130
Luzon	Naga	Bicol Medical Center	134	75	87	321	87	704
Metro Manila	Manila	San Lazaro Hospital	91	272	200	218	215	996
Metro Manila	Valenzuela	Valenzuela Medical Center	57	69	2	1	6	135
		Valenzuela City General Hospital	28	96	38	63	42	267
Metro Manila	Quezon	Quirino Memorial Hospital	80	98	58	404	798	1438
		Quirino Community Hospital	16	35	0	10	0	61
Metro Manila	Muntinlupa	ospital ng muntinlupa	21	57	20	36	30	164
		Research Institute for Tropical Medicine	40	2	65	30	8	145
Visayas	Iloilo	Western Visayas Medical Center	65	122	176	23	59	445
Visayas	Tacloban	Eastern Visayas Regional Medical Center	75	82	337	292	115	901
Mindanao	Surigao	CARAGA Regional Hosptial	447	386	358	111	221	1523
		Surigao Medical Center	0	85	31	0	0	116
Mindanao	Zamboanga	Zamboanga City Medical Center	66	52	153	63	102	436
Mindanao	Cotabato	Cotabato Regional and Medical Center	175	201	229	68	197	870
Mindanao	Davao	Southern Philippines Medical Center	135	252	258	247	243	1135
		Davao Doctors Hospital	53	0	146	0	0	199
**Total**			1548	2964	2478	2111	2805	11906
