# Supplementary material for: Estimating the annual dengue force of infection from the age of reporting primary infections across urban centres in endemic countries
Source: BMC Med. 2021 Sep 30;19:217. doi: 10.1186/s12916-021-02101-6 (PMC8482604; doi:10.1186/s12916-021-02101-6)

S2 File: Population demographics of the study-participating cities

Population demographics of the 13 study participating cities in the Philippines according to the 2015 Philippine census. Source: Philippine Statistics Authority (PSA):

City	Total Population	Median age	Youth DR (<15 years)	Old age DR (>65 years)	% Annual growth rate (2010-15)
Baguio	345366	23.77	47.21	6.13	1.54
Cotabato	299438	21.88	55.62	4.54	1.86
Davao	1632991	24.46	46.31	6.14	2.3
Iliolo	447992	27.08	39.38	8.36	1.03
Manila	1780148	25.37	42.34	5.96	1.43
Muntinlupa	504509	27.68	36.47	5.44	1.78
Naga	196003	24.14	47.16	7.22	2.19
Quezon	2936116	26.39	38.1	5.75	1.17
Surigao	154137	23.91	48.39	7.76	1.77
Tacloban	242089	23.29	48.41	6.5	1.74
Tuguegarao	153502	26.2	37.1	7.19	1.93
Valenzuela	620422	26.09	40.37	5.00	1.45
Zamboanga	861799	23.26	50.98	6.07	1.26

Population age structure of the 13 study-participating cities in the Philippines according to the 2015 Philippine census:

City	Age (years)									Total
	0-4	5-9	10-14	15-19	20-24	25-29	30-34	35-39	>40	
Baguio	37367	34588	34176	36577	36879	29876	25567	22021	88315	345366
Cotabato	34262	35300	34431	34767	29076	24432	20446	20994	65730	299438
Davao	168380	166402	161276	169826	168678	150876	126378	112341	408834	1632991
Iliolo	41946	39230	38238	43482	44468	40014	35486	31488	133640	447992
Manila	186656	164283	157333	177571	191512	170895	144641	123318	463939	1780148
Muntinlupa	44696	43068	41881	45804	50144	49807	50636	37706	140767	504509
Naga	19474	20245	20000	21889	18866	15908	14050	13085	49176	196003
Quezon	260324	259425	257923	296534	315065	282540	245160	219197	799948	2936116
Surigao	15974	15667	16123	17482	15107	12563	10534	9630	41057	154137
Tacloban	26425	24713	24517	28507	25677	20350	17469	14890	59541	242089
Tuguegarao	13462	13267	12739	18100	15949	13433	11730	10733	44089	153502
Valenzuela	55921	58939	57429	60771	63791	61439	54456	49424	158252	620422
Zamboanga	95988	93276	90500	94716	86578	70718	60836	57067	212120	861799

Percentage population age structure of the 13 study-participating cities in the Philippines according to the 2015 Philippine census:

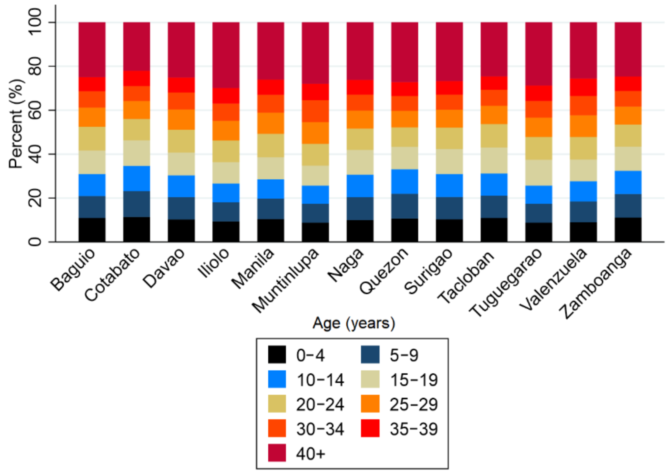

Supplement: Supplementary file 2 — Additional file 2. Population demographics of study-participating cities. [file 12916_2021_2101_MOESM2_ESM.pdf]
