# Supplementary material for: Estimating the annual dengue force of infection from the age of reporting primary infections across urban centres in endemic countries
Source: BMC Med. 2021 Sep 30;19:217. doi: 10.1186/s12916-021-02101-6 (PMC8482604; doi:10.1186/s12916-021-02101-6)
Supplement: Supplementary file 3 — Additional file 3. Methods used to determine primary and post-primary dengue immune status. [file 12916_2021_2101_MOESM3_ESM.pdf]

S3 File: Methods used to determine primary and post-primary dengue immune status

The primary and post-primary immune status of the sampled reporting dengue population with laboratory data was categorised according to a previous developed algorithm. Suspected dengue patients either PCR positive or with IgM panbio units≥9.9 were classified as active dengue infections, while patients PCR negative and with IgM panbio units<9.9 were considered non-active dengue infections. Among active dengue infections, those on disease day 1 or 2 with IgG panbio units above and below 2.2 were categorised as post-primary and primary, respectively. Active cases on disease 3-5, with IgG:IgM ratios above and below 0.45 were classified as post-primary and primary, respectively. Non-active dengue infections were further classified as historical or negative for dengue if they had IgG panbio units above and below 2.2 panbio units, respectively. Post-primary dengue infections include infections with at least one previous flaviviral infection:

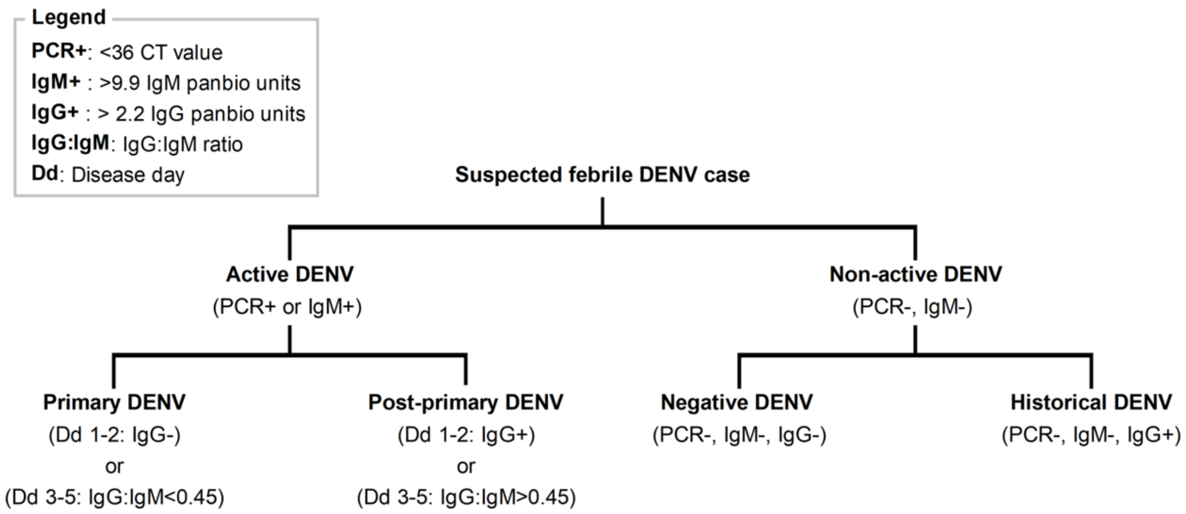

Source: Biggs JR, Sy AK, Brady OJ, *et al.* A serological framework to investigate acute primary and post-primary dengue cases reporting across the Philippines. *BMC Med* 2020; **18**: 364.
