# Supplementary material for: Estimating the annual dengue force of infection from the age of reporting primary infections across urban centres in endemic countries
Source: BMC Med. 2021 Sep 30;19:217. doi: 10.1186/s12916-021-02101-6 (PMC8482604; doi:10.1186/s12916-021-02101-6)
Supplement: Supplementary file 4 — Additional file 4. City-aggregated dengue incidence estimates. [file 12916_2021_2101_MOESM4_ESM.pdf]

S4 File: City-aggregated dengue incidence estimates

City/study period-aggregated all age and age-stratified dengue incidence per annum per 1000 population. Case reports include those who resided and reported in the same city. Incidence equates to total number of cases divided by person years at risk (mean population multiplied by 4 years) multiplied by 1000:

All age dengue incidence:

City	DENV case reports (n)					Population[a] (N)					Incidence pa 1k	
	2014	2015	2016	2017	Total	2014*	2015	2016*	2017*	Mean	Rate	[95%CI]
Baguio	345	1647	2723	512	5227	340047.4	345366	350684.6	356085.2	348045.8	3.75	[3.65-3.86]
Cotabato	492	233	858	201	1784	293868.5	299438	305007.5	310680.7	302248.7	1.48	[1.41-1.54]
Davao	5578	2140	5555	1822	15095	1595432	1632991	1670550	1708972	1651986	2.28	[2.25-2.32]
Iloilo	1216	606	1831	459	4112	443377.7	447992	452606.3	457268.2	450311	2.28	[2.21-2.35]
Manila	1157	3075	2349	2122	8703	1754692	1780148	1805604	1831424	1792967	1.21	[1.19-1.24]
Muntinlupa	340	1153	286	331	2110	495528.7	504509	513489.3	522629.4	509039.1	1.04	[0.99-1.08]
Naga	288	499	341	1097	2225	191710.5	196003	200295.5	204681.9	198172.7	2.81	[2.69-2.92]
Quezon	1260	7163	3815	8716	20954	2901763	2936116	2970469	3005223	2953393	1.77	[1.75-1.80]
Surigao	788	510	918	263	2479	151408.8	154137	156865.2	159641.7	155513.2	3.99	[3.83-4.14]
Tacloban	913	379	106	772	2170	237876.7	242089	246301.3	250587	244213.5	2.22	[2.13-2.31]
Tuguegarao	218	1292	126	568	2204	150539.4	153502	156464.6	159484.4	154997.6	3.55	[3.41-3.70]
Valenzuela	225	358	276	370	1229	611425.9	620422	629418.1	638544.7	624952.7	0.49	[0.46-0.52]
Zamboanga	3837	2671	2990	2253	11751	850940.3	861799	872657.7	883653.2	867262.5	3.39	[3.33-3.45]

Under 5 years dengue incidence:

City	DENV case reports (n)					Population[a] (N)					Incidence pa 1k	
	2014	2015	2016	2017	Total	2014*	2015	2016*	2017*	Mean	Rate	[95%CI]
Baguio	8	38	91	18	155	36791.55	37367	37942.45	38526.77	37656.94	1.03	[0.87-1.19]
Cotabato	54	34	104	30	222	33624.73	34262	34899.27	35548.4	34583.6	1.60	[1.39-1.82]
Davao	586	267	635	213	1701	164507.3	168380	172252.7	176214.6	170338.6	2.50	[2.38-2.62]
Iloilo	102	90	163	74	429	41513.96	41946	42378.04	42814.54	42163.13	2.54	[2.30-2.78]
Manila	95	208	199	169	671	183986.8	186656	189325.2	192032.5	188000.1	0.89	[0.82-0.96]
Muntinlupa	23	75	32	30	160	43900.41	44696	45491.59	46301.34	45097.33	0.89	[0.75-1.02]
Naga	14	16	18	80	128	19047.52	19474	19900.48	20336.3	19689.58	1.63	[1.34-1.91]
Quezon	130	724	555	1222	2631	257278.2	260324	263369.8	266451.2	261855.8	2.51	[2.42-2.61]
Surigao	71	53	103	44	271	15691.26	15974	16256.74	16544.48	16116.62	4.20	[3.70-4.70]
Tacloban	94	79	12	205	390	25965.21	26425	26884.8	27352.59	26656.9	3.66	[3.29-4.02]
Tuguegarao	31	144	19	83	277	13202.18	13462	13721.82	13986.65	13593.16	5.09	[4.49-5.69]
Valenzuela	26	17	20	30	93	55110.15	55921	56731.85	57554.47	56329.37	0.41	[0.33-0.50]
Zamboanga	416	264	317	319	1316	94778.55	95988	97197.45	98422.14	96596.53	3.41	[3.22-3.59]

Under 10 years dengue incidence:

City	DENV case reports (n)					Population[a] (N)					Incidence pa 1k	
	2014	2015	2016	2017	Total	2014*	2015	2016*	2017*	Mean	Rate	[95%CI]
Baguio	52	151	324	62	589	70846.89	71955	73063.11	74188.28	72513.32	2.03	[1.87-2.19]
Cotabato	193	89	422	91	795	68268.15	69562	70855.85	72173.77	70214.94	2.83	[2.63-3.03]
Davao	2002	838	2355	752	5947	327082	334782	342482	350359.1	338676.3	4.39	[4.28-4.50]
Iloilo	512	263	711	239	1725	80339.89	81176	82012.11	82856.84	81596.21	5.29	[5.04-5.53]
Manila	341	869	803	747	2760	345920.6	350939	355957.4	361047.6	353466.2	1.95	[1.88-2.02]
Muntinlupa	83	333	100	125	641	86201.8	87764	89326.2	90916.21	88552.05	1.81	[1.67-1.95]
Naga	34	81	60	343	518	38849.15	39719	40588.85	41477.74	40158.69	3.22	[2.95-3.50]
Quezon	516	2719	1857	4261	9353	513667.9	519749	525830.1	531982.3	522807.3	4.47	[4.38-4.56]
Surigao	281	175	341	119	916	31080.95	31641	32201.05	32771	31923.5	7.17	[6.71-7.64]
Tacloban	327	208	42	523	1100	50248.2	51138	52027.8	52933.08	51586.77	5.33	[5.02-5.65]
Tuguegarao	76	470	55	299	900	26213.13	26729	27244.87	27770.7	26989.42	8.34	[7.79-8.88]
Valenzuela	76	117	88	109	390	113194.5	114860	116525.5	118215.1	115698.8	0.84	[0.76-0.93]
Zamboanga	1441	959	1238	1033	4671	186879.3	189264	191648.7	194063.5	190463.9	6.13	[5.96-6.31]

a: Population according to 2015 Philippine census

*: Estimated population according to city-specific annual growth rates between 2010 & 2015.
