# Supplementary figures and images for: Estimating the annual dengue force of infection from the age of reporting primary infections across urban centres in endemic countries
Source: BMC Med. 2021 Sep 30;19:217. doi: 10.1186/s12916-021-02101-6 (PMC8482604; doi:10.1186/s12916-021-02101-6)

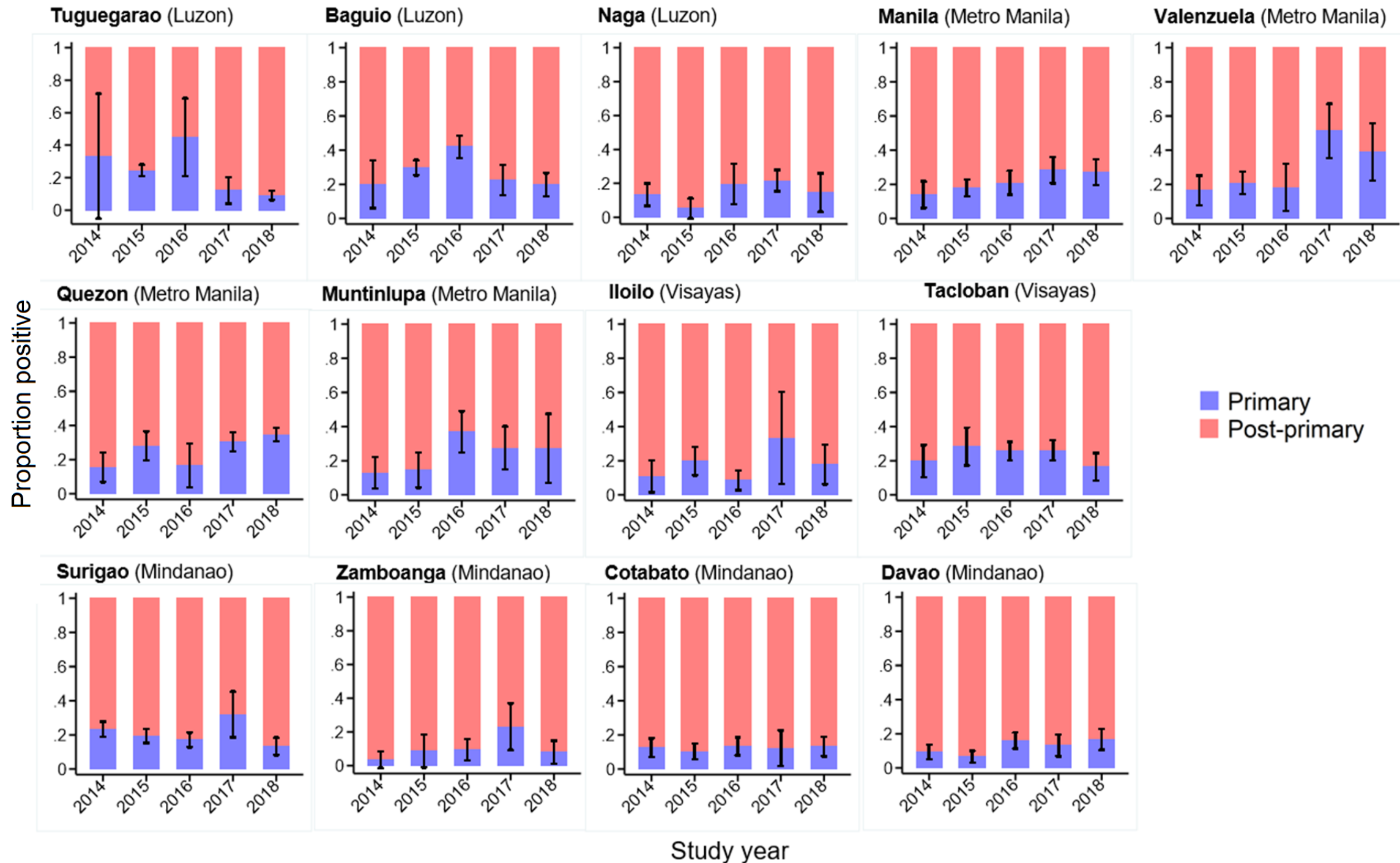

Supplement: Supplementary file 5 — Additional file 5. Reported dengue immune status by year and city. The reported primary/post-primary immune status of reporting active dengue infection by year and city across the Philippines between 2014 and 2018. Vertical bars: 95%CI. [file 12916_2021_2101_MOESM5_ESM.pdf]

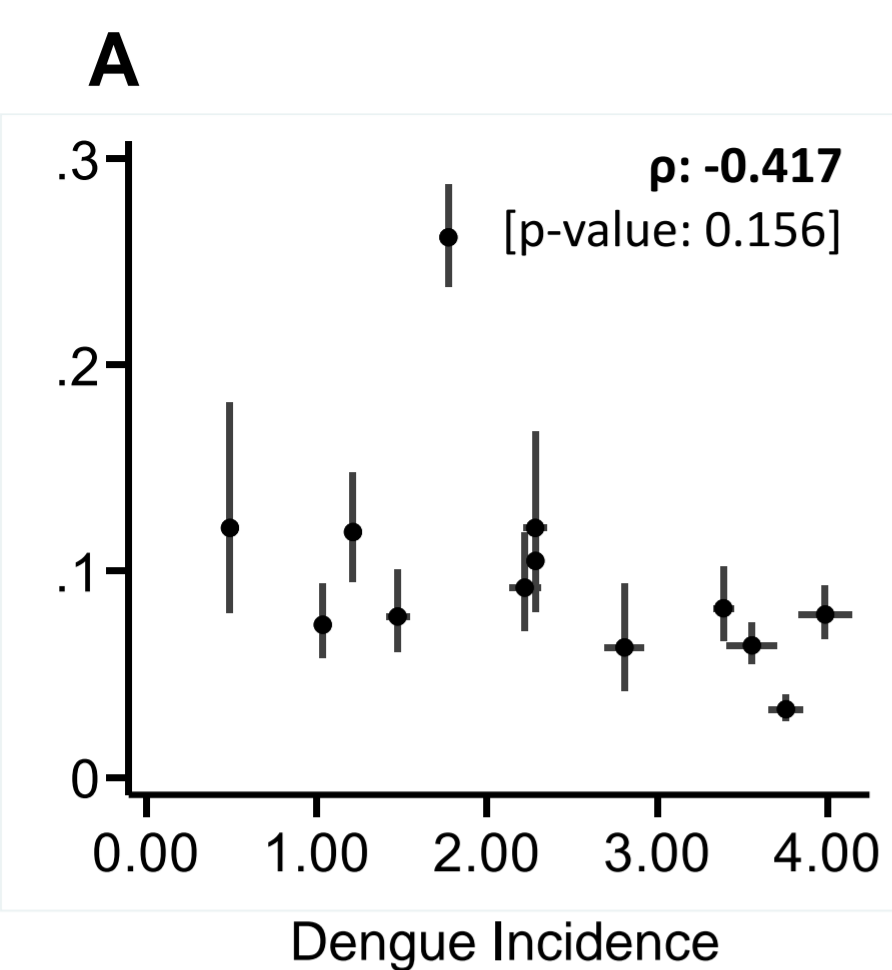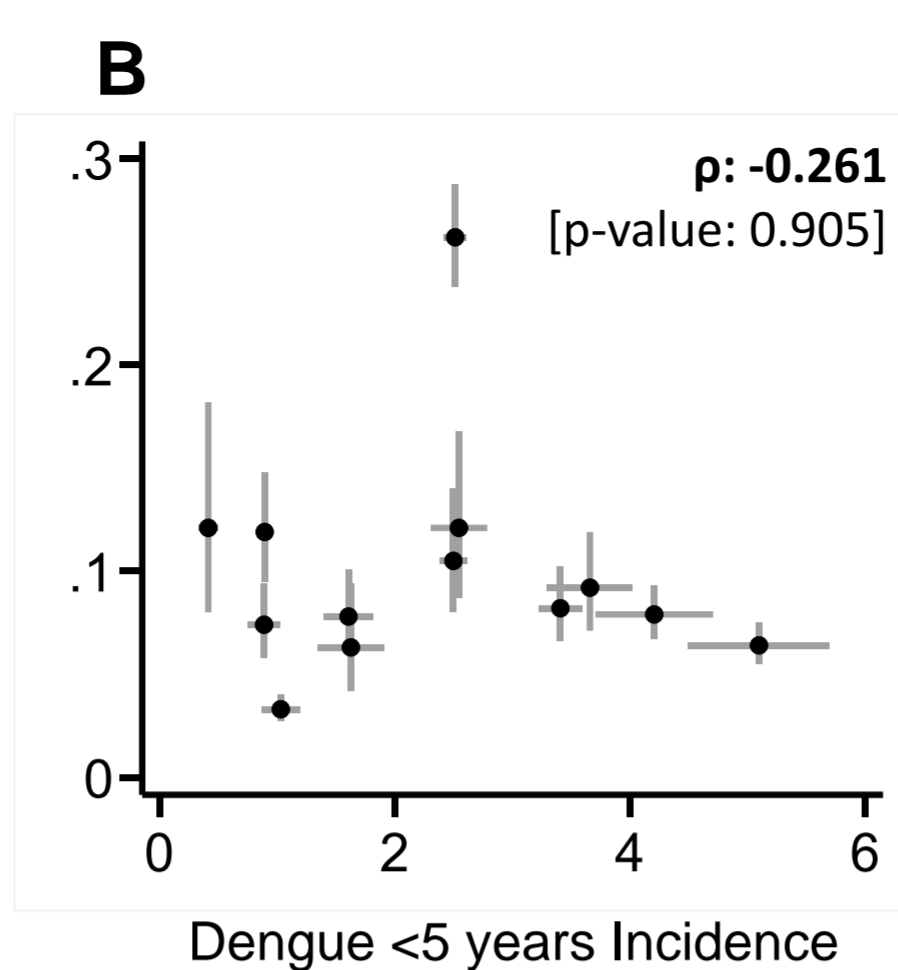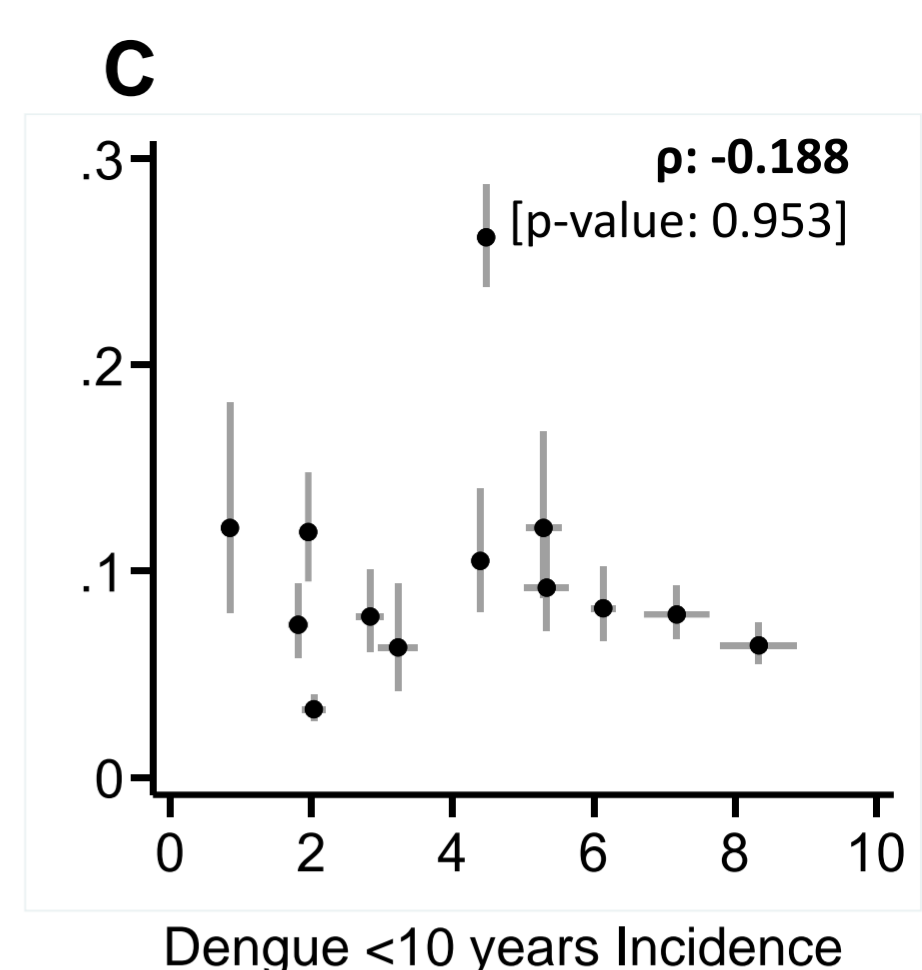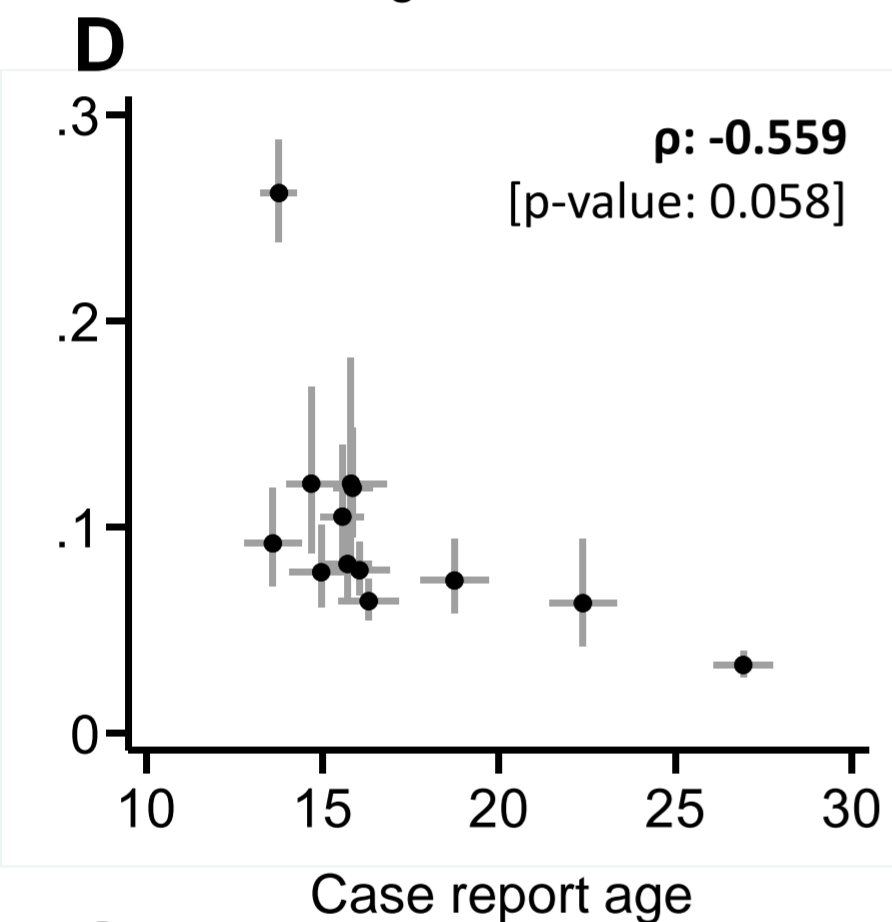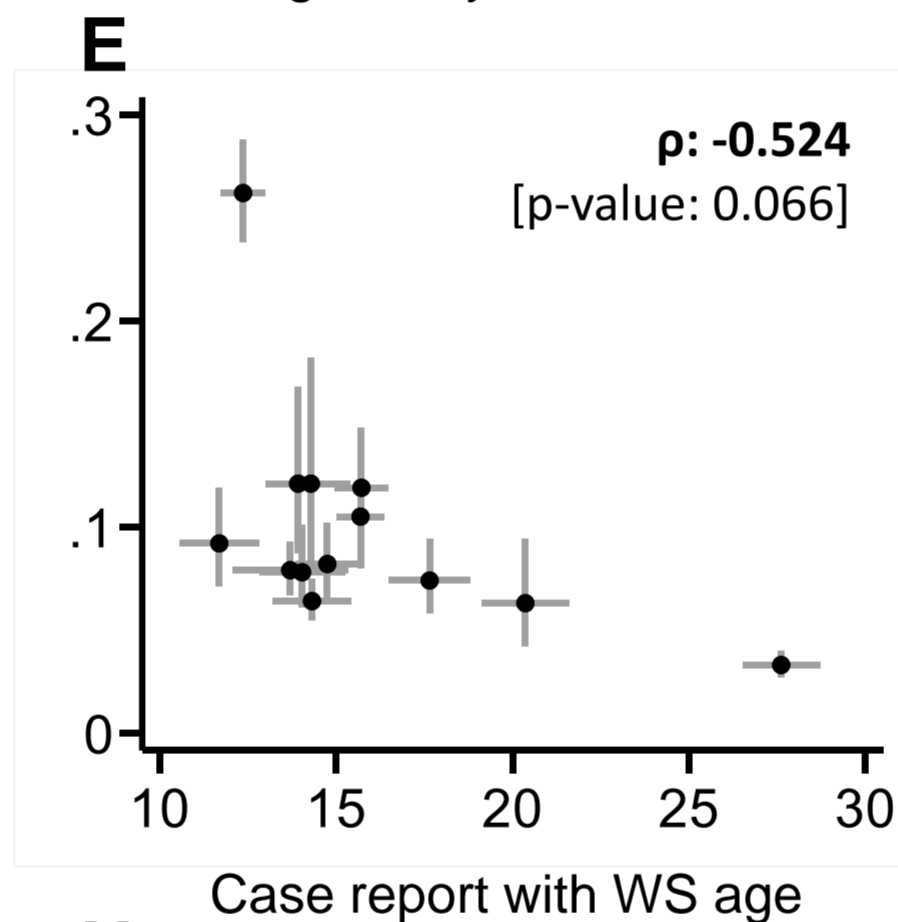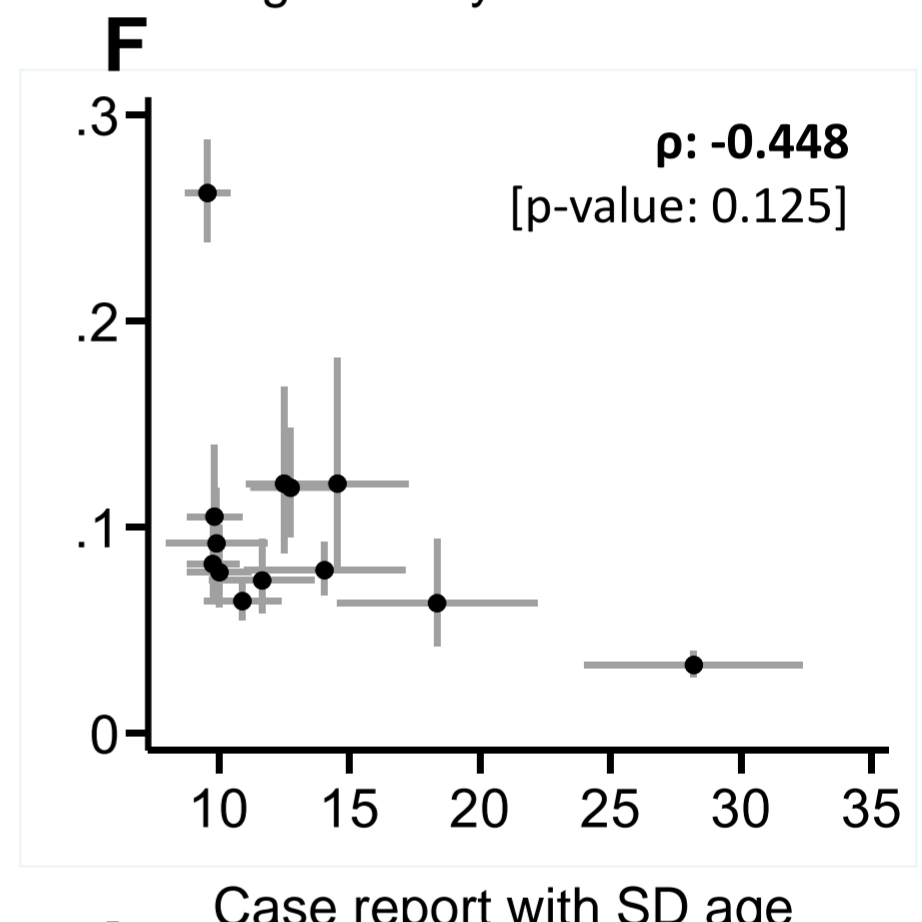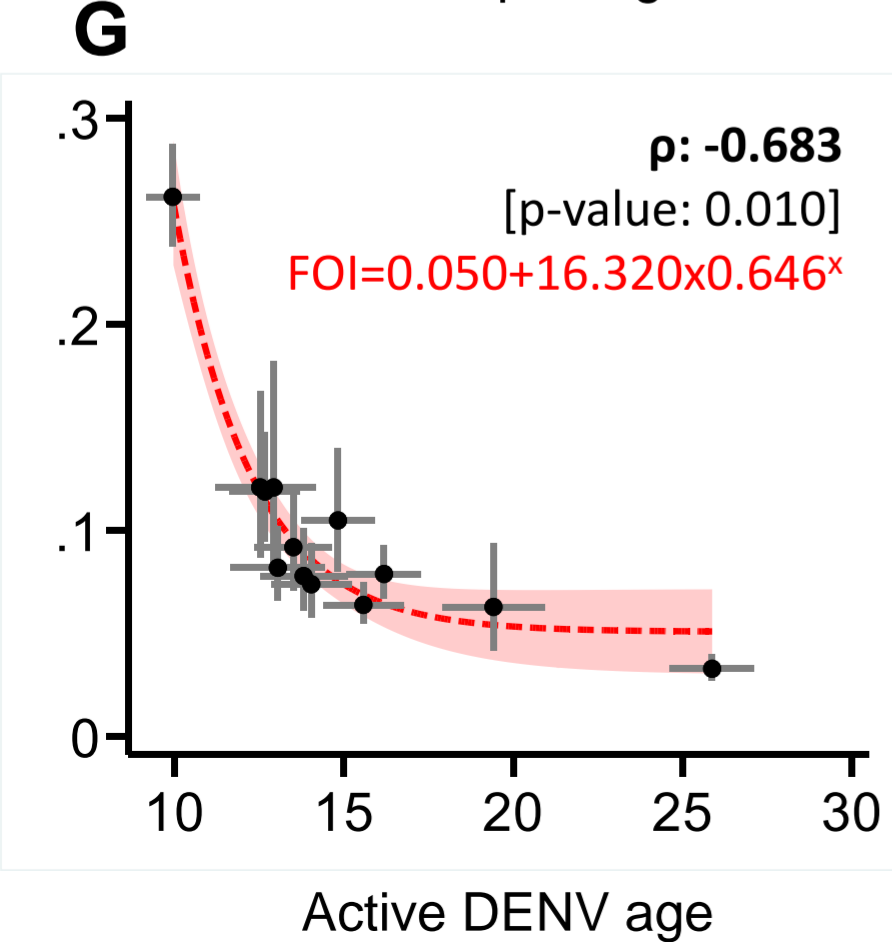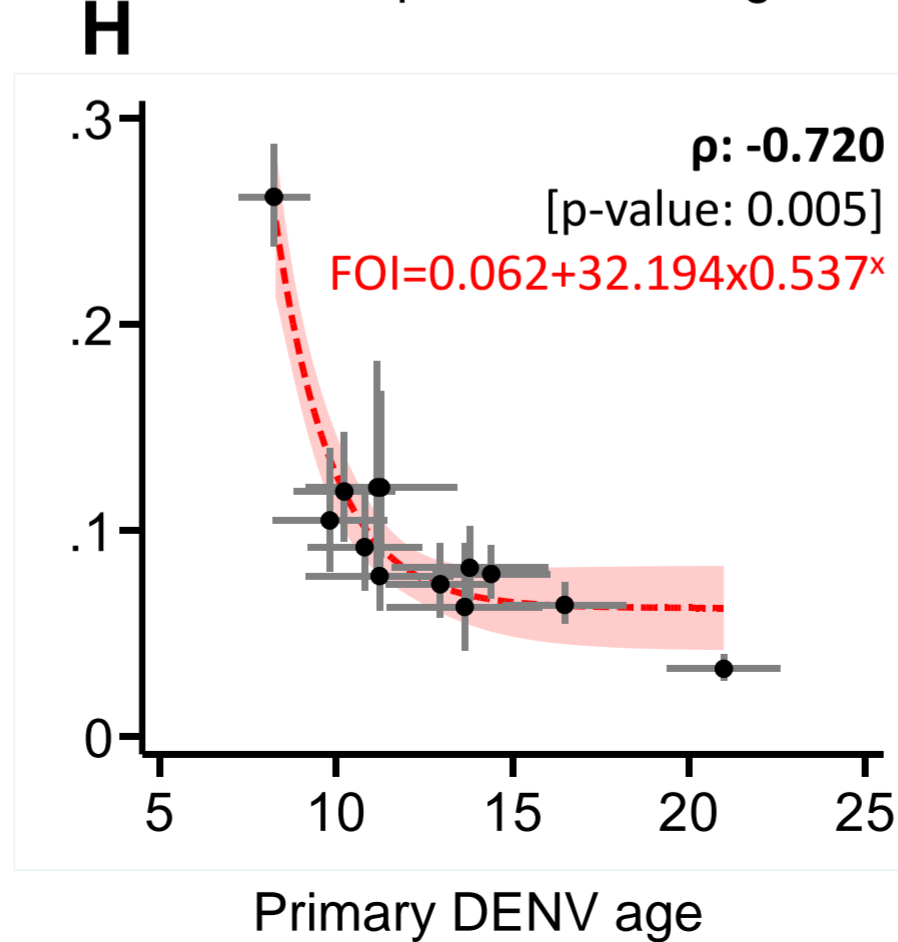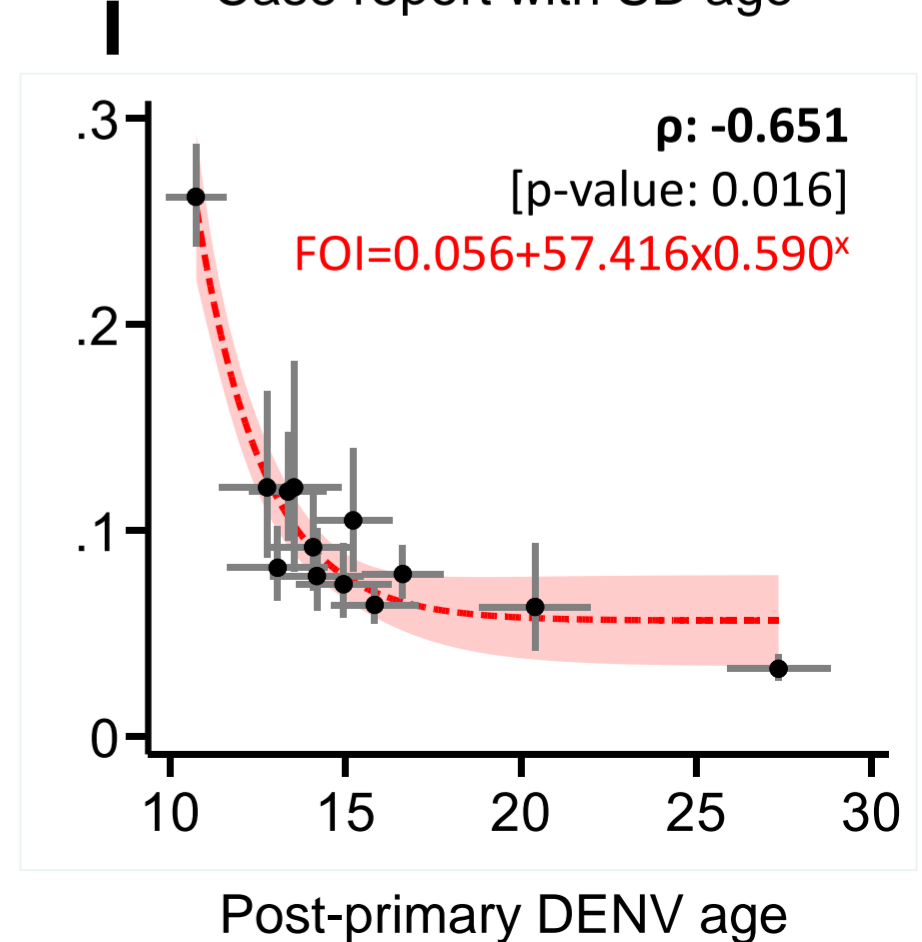

Supplement: Supplementary file 7 — Additional file 7. The city and study period aggregated association between the average annual FOI, according to simple catalytic models, and surveillance metrics. A: crude incidence. B: Under five incidence. C: Under 10 incidence. D: Mean age of case reports. E: Mean age of case reports with warning signs. F: Mean age of case reports with severe dengue. G: Mean age of active infections. H: Mean age of primary dengue infections. I: Mean age of post-primary dengue infections. ρ: Pearson’s R. A-F: Data from passive surveillance G-I: Data from laboratory surveillance. Red dash: predicted FOI according to regression models for metrics with statistically significant associations with FOI (ρ, p-value>0.05). [file 12916_2021_2101_MOESM7_ESM.pdf]
