# Supplementary material for: Estimating the annual dengue force of infection from the age of reporting primary infections across urban centres in endemic countries
Source: BMC Med. 2021 Sep 30;19:217. doi: 10.1186/s12916-021-02101-6 (PMC8482604; doi:10.1186/s12916-021-02101-6)
Supplement: Supplementary file 6 — Additional file 6. FOI catalytic model comparison. Catalytic model fit comparison of simple versus reversible catalytic model used to estimate FOI among sampled cities. AIC: Akaike information criterion. Lower AIC (bold) indicates superior model fit. [file 12916_2021_2101_MOESM6_ESM.pdf]

City	Simple catalytic model			Reverse catalytic model				
	SCR	[95%CI]	AIC	SCR	[95%CI]	SRR	[95%CI]	AIC
Baguio	0.033	[0.027 - 0.04]	268.83	0.054	[0.035 - 0.081]	0.018	[0.007 - 0.048]	**244.30**
Cotabato	0.078	[0.061 - 0.101]	144.35	0.164	[0.126 - 0.203]	0.030	[0.009 - 0.056]	**127.51**
Davao	0.105	[0.08 - 0.14]	131.96	0.178	[0.144 - 0.198]	0.017	[0.005 - 0.035]	**111.40**
Iloilo	0.121	[0.087 - 0.168]	80.31	0.130	[0.096 - 0.175]	<0.001	[0.000 - <0.001]	**78.32**
Manila	0.119	[0.095 - 0.148]	171.75	0.168	[0.154 - 0.187]	0.022	[0.006 - 0.039]	**164.42**
Muntinlupa	0.074	[0.058 - 0.094]	159.38	0.133	[0.094 - 0.196]	0.032	[0.009 - 0.062]	**144.05**
Naga	0.063	[0.042 - 0.094]	129.39	0.063	[0.042 - 0.095]	<0.001	[0.000 - <0.001]	**112.41**
Quezon	0.262	[0.238 - 0.288]	303.29	0.249	[0.223 - 0.279]	<0.001	[0.000 - <0.001]	**291.09**
Surigao	0.079	[0.067 - 0.093]	269.10	0.100	[0.079 - 0.126]	0.006	[0.002 - 0.011]	**251.00**
Tacloban	0.092	[0.071 - 0.119]	168.12	0.121	[0.075 - 0.193]	0.018	[0.002 - 0.047]	**155.25**
Tuguegarao	0.064	[0.055 - 0.075]	142.35	0.067	[0.042 - 0.100]	0.021	[0.004 - 0.056]	**115.11**
Valenzuela	0.121	[0.08 - 0.182]	133.84	0.175	[0.141 - 0.214]	0.029	[0.002 - 0.063]	**118.66**
Zamboanga	0.082	[0.066 - 0.102]	214.52	0.112	[0.051 - 0.176]	0.061	[0.022 - 0.112]	**180.43**
