# Supplementary material for: Estimating the annual dengue force of infection from the age of reporting primary infections across urban centres in endemic countries
Source: BMC Med. 2021 Sep 30;19:217. doi: 10.1186/s12916-021-02101-6 (PMC8482604; doi:10.1186/s12916-021-02101-6)
Supplement: Supplementary file 8 — Additional file 8. Mean annual primary dengue age by city. The average annual age of reported primary dengue infections among study-participating cities between 2014 and 2018. [file 12916_2021_2101_MOESM8_ESM.pdf]

**City**	**Mean primary DENV age (years)**											
	2014		2015		2016		2017		2018		2014-18	
	Mean	[95%CI]	Mean	[95%CI]	Mean	[95%CI]	Mean	[95%CI]	Mean	[95%CI]	Mean	[95%CI]
Baguio	26.6	[22.1-31.1]	25.2	[23.1-27.2]	24.8	[22.5-27.2]	21.9	[18.6-25.1]	16.9	[13.7-20.2]	21.0	[19.4-22.6]
Cotabato	17.1	[13.3-20.8]	12.2	[9.3-15.1]	6.8	[4.7-8.8]	13.0	[10.1-15.9]	9.4	[6.7-12.0]	11.2	[9.1-13.3]
Davao	10.8	[8.4-13.1]	10.8	[8.1-13.5]	9.3	[7.1-11.6]	8.3	[5.7-10.9]	10.3	[8.1-12.5]	9.8	[8.2-11.4]
Iloilo	9.0	[5.7-12.3]	9.8	[7.6-11.9]	8.5	[6.1-10.9]	12.2	[8.7-15.7]	18.3	[14.0-22.5]	11.3	[9.1-13.4]
Manila	8.2	[5.6-10.8]	7.0	[5.3-8.7]	11.0	[8.8-13.2]	10.8	[8.8-12.8]	13.2	[11.1-15.4]	10.2	[8.8-11.7]
Muntinlupa	12.7	[9.6-15.9]	8.1	[5.5-10.8]	9.2	[6.5-12.0]	16.9	[13.3-20.4]	13.5	[10.5-16.5]	13.0	[11.4-14.5]
Naga	22.2	[17.9-26.5]	11.3	[7.5-15.0]	9.2	[6.0-12.3]	12.6	[10.0-15.2]	13.8	[11.0-16.7]	13.6	[11.4-15.8]
Quezon	9.6	[7.2-12.1]	7.9	[5.8-10.0]	8.6	[5.0-12.2]	8.4	[7.0-9.8]	8.2	[7.0-9.3]	8.2	[7.2-9.3]
Surigao	13.2	[11.1-15.3]	14.5	[12.1-16.8]	15.1	[12.6-17.6]	12.4	[9.0-15.8]	18.4	[15.5-21.3]	14.4	[12.7-16.1]
Tacloban	15.5	[12.0-19.0]	10.9	[7.7-14.1]	8.6	[6.8-10.4]	8.9	[7.0-10.9]	9.8	[7.4-12.2]	10.8	[9.2-12.4]
Tuguegarao	10.6	[6.2-14.9]	15.1	[13.1-17.1]	11.4	[8.7-14.2]	12.5	[9.6-15.4]	13.8	[11.3-16.3]	13.3	[12.1-14.5]
Valenzuela	10.8	[7.9-13.6]	11.3	[9.2-13.5]	9.2	[5.7-12.6]	11.9	[9.6-14.2]	10.9	[8.9-12.9]	11.2	[9.5-12.8]
Zamboanga	27.5	[26.1-28.9]	13.7	[8.5-18.8]	10.5	[7.5-13.6]	9.0	[6.0-12.0]	6.4	[3.7-9.1]	13.8	[11.6-16.0]
