# Supplementary material for: Estimating the annual dengue force of infection from the age of reporting primary infections across urban centres in endemic countries
Source: BMC Med. 2021 Sep 30;19:217. doi: 10.1186/s12916-021-02101-6 (PMC8482604; doi:10.1186/s12916-021-02101-6)
Supplement: Supplementary file 9 — Additional file 9. Mean annual age of cases with dengue warning signs by city. The average annual age of reported dengue cases with warning signs among study-participating cities between 2014 and 2018. [file 12916_2021_2101_MOESM9_ESM.pdf]

City	Mean age of dengue cases with warning signs (years)											
	2014		2015		2016		2017		2018		2014-18	
	Mean	[95%CI]	Mean	[95%CI]	Mean	[95%CI]	Mean	[95%CI]	Mean	[95%CI]	Mean	[95%CI]
Tuguegarao	11.0	[7.1-14.9]	15.1	[13.8-16.4]	11.8	[9.4-14.2]	11.1	[9.2-13.0]	18.1	[16.5-19.7]	16.3	[15.1-17.5]
Baguio	21.4	[17.6-25.2]	29.8	[27.2-32.5]	29.0	[26.7-31.2]	24.2	[21.7-26.6]	22.3	[19.7-24.9]	25.4	[23.5-27.2]
Valenzuela	12.9	[11.2-14.7]	11.5	[10.1-12.9]	15.2	[12.9-17.6]	12.6	[10.9-14.3]	15.1	[12.7-17.5]	12.7	[11.4-14.0]
Quezon	12.6	[10.5-14.7]	10.7	[9.1-12.4]	15.5	[13.0-18.1]	9.4	[8.4-10.4]	9.5	[8.6-10.5]	9.9	[9.1-10.7]
Manila	10.2	[8.6-11.7]	10.8	[9.6-12.0]	12.5	[11.0-14.0]	14.2	[12.6-15.7]	15.7	[14.1-17.2]	13.1	[12.1-14.2]
Muntinlupa	13.0	[9.9-16.1]	16.4	[13.6-19.1]	12.2	[10.1-14.4]	14.5	[12.4-16.7]	14.8	[12.0-17.7]	13.9	[12.2-15.6]
Naga	22.7	[20.4-24.9]	21.0	[18.5-23.4]	19.9	[17.3-22.4]	18.4	[16.5-20.4]	13.4	[11.5-15.4]	18.2	[16.6-19.8]
Iloilo	9.9	[8.1-11.6]	10.6	[9.2-12.0]	12.8	[11.3-14.3]	8.4	[6.3-10.5]	15.8	[13.5-18.1]	12.4	[11.1-13.6]
Tacloban	25.0	[19.6-30.4]	15.1	[11.7-18.5]	11.6	[10.1-13.1]	11.5	[9.0-14.1]	12.5	[10.0-15.0]	11.9	[10.4-13.3]
Surigao	12.5	[10.5-14.5]	18.8	[15.3-22.3]	12.3	[8.4-16.1]	15.8	[12.8-18.8]	13.3	[11.2-15.5]	18.0	[14.7-21.3]
Davao	12.7	[11.0-14.3]	14.8	[13.0-16.6]	14.0	[12.5-15.4]	16.1	[14.3-18.0]	11.9	[10.5-13.2]	14.8	[13.6-16.0]
Cotabato	13.6	[11.3-16.0]	16.9	[14.9-18.8]	12.0	[10.3-13.7]	18.5	[15.6-21.4]	13.6	[11.8-15.5]	15.5	[14.0-17.0]
Zamboanga	16.6	[14.1-19.1]	11.2	[8.5-13.8]	12.4	[10.8-14.0]	10.3	[8.7-11.9]	11.0	[9.1-12.8]	11.8	[10.4-13.1]
