# Supplementary material for: Estimating the annual dengue force of infection from the age of reporting primary infections across urban centres in endemic countries
Source: BMC Med. 2021 Sep 30;19:217. doi: 10.1186/s12916-021-02101-6 (PMC8482604; doi:10.1186/s12916-021-02101-6)
Supplement: Supplementary file 10 — Additional file 10. Annual city Attack rates by city according to the mean age of suspected dengue cases with warning signs. [file 12916_2021_2101_MOESM10_ESM.pdf]

City	Predicted annual attack rate*a						Attack rate (AR) (catalytic model)
	2014	2015	2016	2017	2018	2014-18 average	
	AR [95%CI]	AR [95%CI]	AR [95%CI]	AR [95%CI]	AR [95%CI]	AR [95%CI]	AR [95%CI]
Tuguegarao	0.19 [0.13-0.26]	0.13 [0.12-0.15]	0.17 [0.14-0.22]	0.18 [0.16-0.22]	0.10 [0.09-0.12]	0.12 [0.11-0.13]	0.06 [0.04-0.10]
Baguio	0.08 [0.06-0.10]	0.04 [0.04-0.05]	0.05 [0.04-0.05]	0.06 [0.05-0.08]	0.07 [0.06-0.09]	0.06 [0.05-0.07]	0.05 [0.03-0.08]
Valenzuela	0.16 [0.13-0.18]	0.18 [0.16-0.20]	0.13 [0.10-0.16]	0.16 [0.14-0.19]	0.13 [0.11-0.16]	0.16 [0.14-0.18]	0.16 [0.13-0.19]
Quezon	0.16 [0.13-0.20]	0.19 [0.16-0.22]	0.13 [0.10-0.16]	0.22 [0.20-0.24]	0.21 [0.20-0.23]	0.21 [0.19-0.22]	0.22 [0.20-0.24]
Manila	0.20 [0.18-0.23]	0.19 [0.17-0.21]	0.16 [0.14-0.19]	0.14 [0.12-0.16]	0.12 [0.11-0.14]	0.15 [0.14-0.17]	0.15 [0.14-0.17]
Muntinlupa	0.16 [0.12-0.21]	0.12 [0.09-0.15]	0.17 [0.14-0.20]	0.14 [0.11-0.16]	0.13 [0.10-0.17]	0.14 [0.12-0.17]	0.12 [0.09-0.18]
Naga	0.07 [0.06-0.08]	0.08 [0.07-0.10]	0.09 [0.07-0.11]	0.10 [0.08-0.12]	0.15 [0.13-0.18]	0.10 [0.09-0.11]	0.06 [0.04-0.09]
Iloilo	0.21 [0.18-0.24]	0.19 [0.17-0.22]	0.16 [0.14-0.18]	0.24 [0.20-0.28]	0.12 [0.10-0.15]	0.17 [0.15-0.19]	0.12 [0.09-0.16]
Tacloban	0.06 [0.04-0.09]	0.13 [0.10-0.18]	0.18 [0.15-0.20]	0.18 [0.14-0.22]	0.16 [0.13-0.20]	0.17 [0.15-0.20]	0.11 [0.07-0.18]
Surigao	0.16 [0.14-0.20]	0.09 [0.07-0.13]	0.17 [0.12-0.24]	0.12 [0.09-0.16]	0.15 [0.13-0.18]	0.10 [0.08-0.13]	0.10 [0.08-0.12]
Davao	0.16 [0.14-0.19]	0.13 [0.11-0.16]	0.14 [0.13-0.16]	0.12 [0.10-0.14]	0.17 [0.15-0.20]	0.13 [0.12-0.15]	0.16 [0.13-0.18]
Cotabato	0.15 [0.12-0.18]	0.11 [0.09-0.13]	0.17 [0.15-0.20]	0.10 [0.08-0.12]	0.15 [0.13-0.17]	0.13 [0.11-0.14]	0.15 [0.12-0.18]
Zamboanga	0.11 [0.09-0.14]	0.18 [0.15-0.23]	0.17 [0.14-0.19]	0.20 [0.17-0.23]	0.19 [0.16-0.22]	0.17 [0.15-0.20]	0.11 [0.05-0.16]

*Attack rate (AR): 1-exp$^{(-FOI)}$

a: Estimated annual attack rate according to mean primary DENV age (FOI=0.023+0.630x0.852^{x})

b: Estimated overall attack rate according to FOI estimated from catalytic model

Attack rate:	
0.25-0.30	
0.20-0.25	
0.15-0.20	
0.10-0.15	
0.05-0.10	
0.05-0.10	
